# Organoids simulating the bovine oviduct mediate the embryo–maternal interface via extracellular vesicle-transmitted signaling

**DOI:** 10.1093/hropen/hoaf076

**Published:** 2025-12-05

**Authors:** Nico G Menjivar, Ahmed Gad, Riley E Thompson, Mindy A Meyers, Soham Ghosh, Fiona K Hollinshead, Dawit Tesfaye

**Affiliations:** Animal Reproduction and Biotechnology Laboratory (ARBL), Department of Biomedical Sciences, College of Veterinary Medicine and Biomedical Sciences, Colorado State University, Fort Collins, CO, USA; Stanford Fertility and Reproductive Health Services, Stanford Medicine Children’s Health, Sunnyvale, CA, USA; Animal Reproduction and Biotechnology Laboratory (ARBL), Department of Biomedical Sciences, College of Veterinary Medicine and Biomedical Sciences, Colorado State University, Fort Collins, CO, USA; Department of Animal Production, Faculty of Agriculture, Cairo University, Giza, Egypt; Animal Reproduction and Biotechnology Laboratory (ARBL), Department of Clinical Sciences, College of Veterinary Medicine and Biomedical Sciences, Colorado State University, Fort Collins, CO, USA; Animal Reproduction and Biotechnology Laboratory (ARBL), Department of Clinical Sciences, College of Veterinary Medicine and Biomedical Sciences, Colorado State University, Fort Collins, CO, USA; Cellular Engineering and Mechanobiology Laboratory (CEML), Translational Medicine Institute (TMI), Department of Mechanical Engineering, Colorado State University, Fort Collins, CO, USA; Animal Reproduction and Biotechnology Laboratory (ARBL), Department of Clinical Sciences, College of Veterinary Medicine and Biomedical Sciences, Colorado State University, Fort Collins, CO, USA; Animal Reproduction and Biotechnology Laboratory (ARBL), Department of Biomedical Sciences, College of Veterinary Medicine and Biomedical Sciences, Colorado State University, Fort Collins, CO, USA

**Keywords:** organoids, extracellular vesicles, oviduct, heat stress, miRNA

## Abstract

**STUDY QUESTION:**

Does the implementation of a three-dimensional (3D) organoid model system that stably emulates some key functional, structural, and biological complexities of the oviduct provide a favorable apical environment for the production of extracellular vesicles (EVs) that exert an influence on early embryo development *in vitro*?

**SUMMARY ANSWER:**

Our findings show that *in vitro*, epithelium dependably propagates highly differentiated oviductal organoids containing both networks of ciliated and secretory cells capable of producing *in vivo*-like, cargo-specific oviductal extracellular vesicles (oEVs) with the capacity to improve the quality of *in vitro*-produced embryos under conditions of heat stress (HS).

**WHAT IS KNOWN ALREADY:**

Recapitulating the maternal contribution that persists during preimplantation embryonic development *in vitro* is a substantial scientific challenge due to both technical limitations and the significant gaps in our scientific knowledge concerning the maternal–embryonic cellular and molecular dialogue. As a result of the limited access to suitable model systems and the inability to directly observe this process *in vivo*, this early stage of embryonic development has often been described as particularly elusive and an enigmatic stage of development. Irrespectively, oEVs have recently been identified as key players in mediating the biological information transfer of the embryo–oviduct interactions, which beneficially contributes to the early development of embryos *in vitro*.

**STUDY DESIGN, SIZE, DURATION:**

Over a 2-year period, resected ovaries from intact reproductive tracts (n = 10; a pool of two bovine animals per replicate) containing both complete contralateral and ipsilateral oviducts from assessed stage II, diestrus tracts were processed for the generation of oviductal organoids. Afterward, enriched oEVs from 3D organoids and *in vivo*-collected oviductal fluid (OF) were co-cultured with bovine presumptive zygotes from Day 1 to Day 3 and continued until the blastocyst stage for further evaluation.

**PARTICIPANTS/MATERIALS, SETTING, METHODS:**

Organoids were characterized by light microscopy, gene expression, immunofluorescence, and 3D reconstruction, as well as histological two-dimensional (2D) cross-sectioning. Enriched oEVs from conditioned organoid culture media and OF were characterized using transmission electron microscopy, nanoparticle tracking analysis, and western blotting. Following the establishment of a stable oEV production system, bovine zygotes were divided into five groups [38.5°C Control, 41°C Control, 41°C N-EVs (oEVs derived from organoids cultured under thermoneutral conditions), 41°C S-EVs (oEVs derived from organoids cultured HS conditions), 41°C Ovi-EVs (oEVs collected from diestrus OF)] and cultured until the blastocyst stage. Following the presence or absence of oEVs during Day 1 to Day 3 of *in vitro* culture, the resulting cleavage and blastocyst developmental rates were recorded. We also conducted co-immunostaining for trophectoderm (CDX2) and inner cell mass (SOX2) pluripotency marker proteins, detected global DNA damage (phospho-γH2A.X), and performed real-time quantitative PCR assays in individual embryos for candidate embryo quality genes *CDX2*, *SOX2, POU5F1*, *NANOG*, and critical stress-regulating genes *BAX*, *BCL-2, PRDX1*, *SOD1, HSP70*, and *HSP90*. Additionally, the influence of oEVs on the epigenetic landscapes of developing embryos was analyzed through their perturbations to H3K9ac, and competitive marks H3K27ac and H3K27me3, in association to their relative expressions of hallmark DNA methyltransferases (*DNMT1*, *DNMT3A*, *DNMT3B*) among individual embryos.

**MAIN RESULTS AND THE ROLE OF CHANCE:**

Here, we employed a 3D culture system to generate oviductal organoids to mimic the maternal environment’s response to HS and for the production of *in vivo*-like oEVs, which were used to enhance the survival and viability of *in vitro*-produced embryos under conditions of stress. Interestingly, our findings also effectively demonstrate the first attempt at underpinning emerging parallels in EV-packaged miRNAs released from 3D oviductal organoids, 2D oviductal epithelial cells, and *in vivo*-collected oEVs persistently present within OF. The aim of this approach sustains a mechanistic alternative in robustly generating physiologically relevant oEVs to improve the current *in vitro* culture system, which traditionally bypasses the oviduct. This model system also innovatively enhances our knowledge of the EV-mediated, maternal-embryonic communication occurring *in vivo*.

**LARGE SCALE DATA:**

N/A.

**LIMITATIONS, REASONS FOR CAUTION:**

This was an *in vitro* study in which conditions of the organoid cultures may not exactly mirror the *in vivo* environment in terms of the oviducts’ extracellular matrix and complex vascularization. Additionally, given the polarity of the 3D organoids utilized within this study, the population of enriched oEVs largely represents basolateral secretions versus the conventional apical secretions *in vivo*.

**WIDER IMPLICATIONS OF THE FINDINGS:**

These results provide an uncharted attempt at recapitulating embryo–maternal nano-communication through the means of oEVs secreted from 3D oviductal organoids cultured *ex vivo*. Thus, our model establishes a foundation for incorporating oviductal cues that modulate embryonic development *in vitro*, providing a dynamic system to further investigate mechanisms by which the maternal environment may contribute to the early successes of embryonic development and, offering valuable insights that could facilitate advancements in current *in vitro* embryo production technologies.

**STUDY FUNDING/COMPETING INTEREST(S):**

This study was supported by the United States Department of Agriculture through a NIFA-AFRI Predoctoral Fellowship awarded to N.G.M. (Grant Number 2023-67011-40511), as well as funds from the College Research Council, Office of the Vice President for Research at Colorado State University. The authors attest that there are no competing interests that could have influenced the conduct or outcomes of this research.

WHAT DOES THIS MEAN FOR PATIENTS? Outside the natural environment of the uterus, the first 7 days of embryo development underscore a finely tuned balance between the embryo’s instinctive capacity for self-organization and the precisely controlled laboratory environment that supports it, despite the fact that current standards retain the immense rate-limiting feature of innate embryo–maternal communication. By creating a three-dimensional model of the oviduct (fallopian tube), known through previous studies to produce biologically relevant maternal signals in the form of extracellular vesicles (EVs), we successfully recreated, in the laboratory, some of the natural signaling molecules that would normally support early embryo development *in vivo*, completely *in vitro*. In our study, these oEVs aided the ability of embryos to cope with heat stress and improved their overall developmental quality. For patients, this research serves as a step toward developing IVF culture conditions that more closely resemble the natural reproductive environment. If future studies, including those using human-derived tissues, show similar benefits, integrating these physiological cues into standard embryo culture conditions could one day enhance embryo resilience, development, and clinical IVF outcomes.

## Introduction

Essential for fertility, the oviducts (anatomically synonymous to the fallopian tubes in women) are bilateral conduits segmented into three main regions (infundibulum, ampulla, and isthmus), extending from the ovaries to the uterus. Although comprising a highly dynamic and well-orchestrated microenvironment, originally the oviducts were acknowledged less for their benefit in embryo development and physiology and more as a means of delaying the transport of the embryo into the uterus preceding the initiation of a proliferative and receptive endometrium under effective hormonal regulation (Hunter, 1998). Ultimately, the importance of the oviducts has long been contended given that embryos can be produced *in vitro* via the bypassing of the maternal environment and supported through the initial stages of germinal development. However, a progressive understanding of the roles of oviducts spanning the most recent decades has continued to shed light upon and reveal their involvement in a multitude of principal processes (gamete maturation, sperm capacitation, sperm selection and storage, early embryo development, genome activation, etc.) essential to fertility and embryo developmental competence in particular (Avilés *et al.*, 2010). *In vivo*, the bovine oviduct undergoes imperative variation in spatial transcriptional differences during periodic cyclical changes, largely regulated by fluctuating ovarian steroid hormones (E2 and P4) (Saint-Dizier *et al.*, 2012; Cerny *et al.*, 2015; Gonella-Diaza *et al.*, 2015; Maillo *et al.*, 2016a). In this regard, the oviductal epithelium lining the hollow seromuscular organ is responsible for propagating the dynamic secretions comprising oviductal fluid (OF), central to establishing an optimal environmental milieu for gamete maturation, fertilization, and the early cleavage of developing embryos (Hunter, 2012). The indispensable role of the oviductal milieu in shaping the future embryo has been previously demonstrated through the culture of *in vitro*-produced bovine zygotes, 4-cell and 8-cell embryos, and morulae amidst the oviduct of cattle *in vivo*, revealing modulations to the transcriptome (Gad *et al.*, 2012) and methylome of the resulting blastocysts (Salilew-Wondim *et al.*, 2015). Unlike the ovary and the uterus, which has been extensively studied and is relatively well understood, attempts to better understand embryo–maternal interactions during early stages of development is a substantial scientific challenge due to its technical inaccessibility and ethical concerns, as well as the lack of suitable *in vitro* model systems, all of which further highlight the significance of propagating relevant models to recapitulate aspects of preimplantation embryo–maternal communication.

Regarding the bovine model, once fertilized, the resulting zygote traditionally spends its first 3–4 days post-fertilization within the oviduct, wherein, it undergoes several critical developmental milestones (Memili and First, 2000; Croxatto, 2002; Li and Winuthayanon, 2017), specifically, the undertaking of gametic syngamy and the first cleavage cell divisions that precede the maternal-to-zygotic transition (MZT), mechanisms by which the embryo assumes self-sufficiency in order to undergo both minor (Ram and Schultz, 1993; Abe *et al.*, 2018) and major embryonic genome activations (EGA; at the 8- to 16-cell stage), resulting in the low to wide-spread transcription of embryonic genes, echoing a strong similarity to events observed in humans (Braude *et al.*, 1988; Schulz and Harrison, 2019; Halstead *et al.*, 2020). *In vivo*, OF, consisting of a plethora of energy substrates (Hugentobler *et al.*, 2008; Lamy *et al.*, 2018), lipids (Belaz *et al.*, 2016; Jordaens *et al.*, 2017; Banliat *et al.*, 2019), and various proteins/embryotrophic factors (Lamy *et al.*, 2016; Pillai *et al.*, 2017; Papp *et al.*, 2019), as well as oviductal extracellular vesicles (oEVs) (Maillo *et al.*, 2016b,c; Almiñana and Bauersachs, 2019). More recently, oEVs have been identified to serve as a mass transit mechanism as pertinent communicative (Théry, 2011) RNA nanoshuttles (Valadi *et al.*, 2007; Menjivar *et al.*, 2024), responsible for mediating the early embryo–maternal interactions. Nevertheless, oviduct-secreted factors comprising the OF actively combat physical stress from the environment (Christians *et al.*, 1995, 1997; Mariani *et al.*, 2000, 2003; Le Masson and Christians, 2011) and various forms of oxidative stress (Guérin *et al.*, 2001) to ensure embryo quality and pregnancy outcomes. Until now, exhaustive measures *in vitro* to probe OF and model aspects of the embryo-epithelial interface have predominantly employed two-dimensional (2D) monolayers of oviductal epithelium (Thibodeaux *et al.*, 1991; Walter, 1995). Regarding the former, in this orientation, oviduct epithelial cells (OECs) function to respond to exogenous hormonal stimulation (Rottmayer *et al.*, 2006; Simintiras *et al.*, 2016), reclaim ciliation (Chen *et al.*, 2017), and compositionally express hallmark oviduct-specific glycoproteins (Simintiras *et al.*, 2016; Chen *et al.*, 2017), ultimately supporting early embryo development (Chen *et al.*, 2017). While a cost-effective platform, 2D cell cultures contain inherent drawbacks with critical restrictions in the forfeiture of *in vivo* tissue-like structures and long-term physiological function considering their rapid loss of typical differentiated OEC properties, such as ciliation, columnar cell morphology, cell polarity, and secretory capacity (Joshi, 1995; Schoen *et al.*, 2008; Ulbrich *et al.*, 2010; Gualtieri *et al.*, 2012; Karst and Drapkin, 2012).

**Figure 1.**
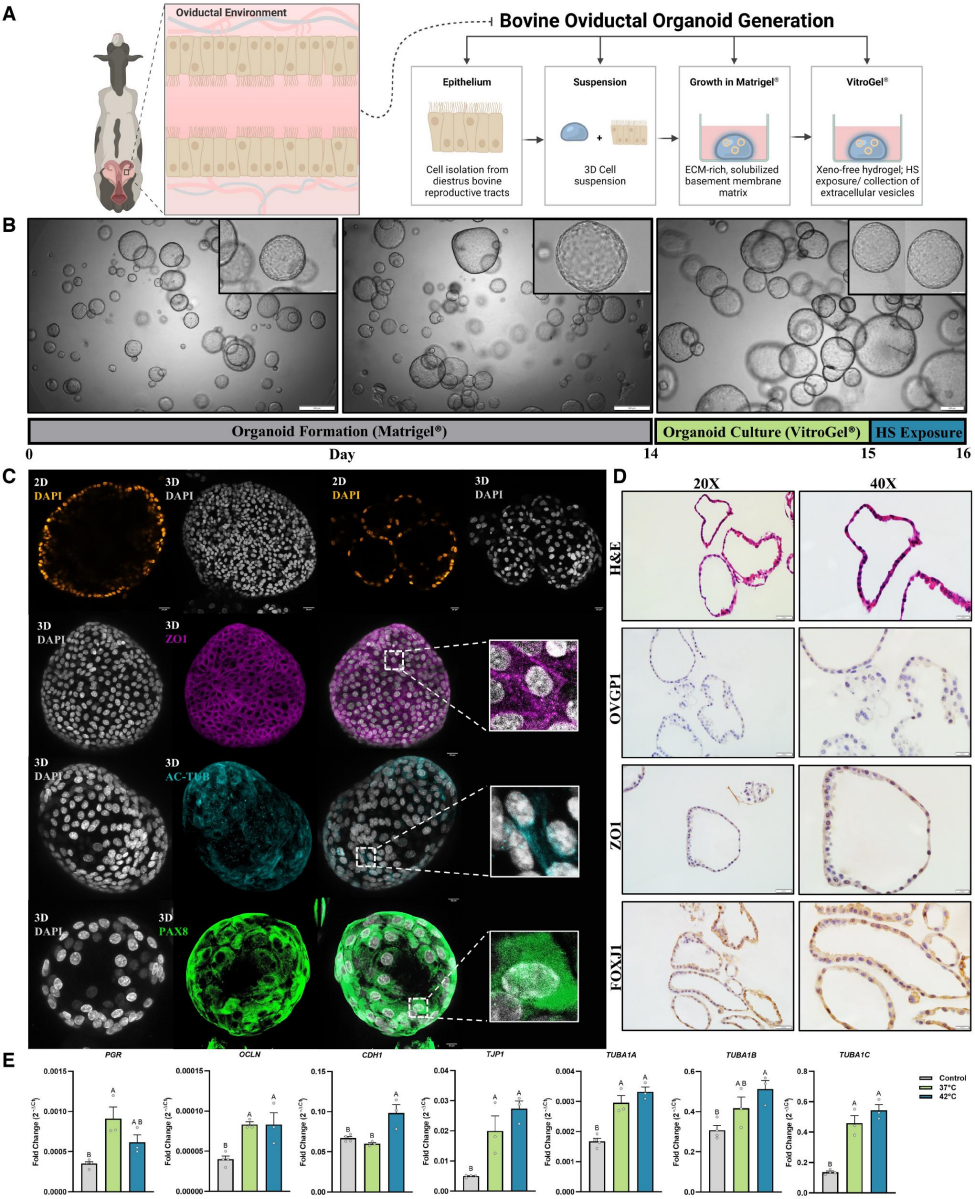
**Establishment and characterization of bovine oviductal epithelial organoids**. (**A**) Illustration of the anatomical tube-like passageway (oviduct) in the female reproductive tract responsible for harboring the source cells used for the generation of oviductal organoids in the schematic representation of the culture method. (**B**) Gross morphology, bright-field organoid images. Scale bars, 200 or 500 µm (magnified inserts 50 or 100 µm). (**C**) Round, cystic organoid structures formed under specific culture conditions as described in the Materials and Methods section express Zonula Occludens-1 (ZO1; pseudo-colored magenta), acetylated tubulin (AC-Tub; pseudo-colored cyan), and PAX8, counterstained with DAPI (pseudo-colored orange hot in 2D cross-sections and gray in 3D z-projections) for nuclei visualization. (**D**) Histological, sectioned (6 µm) oviductal organoids with H&E staining, and immunohistochemical (OVGP1, ZO1, FOXJ1) at 20× (Scale bar, 50 μm) and 40× magnification (Magnified insert; Scale bar, 20 μm), respectively. (**E**) Relevant gene panel of *in vitro*-produced organoid retention of the *in vivo* oviduct. Relative *PGR*, *OCLN*, *CDH1*, *TJP1*, *TUBA1A*, *TUBA1B*, and *TUBA1C* mRNA levels in non-hormone-stimulated [control, thermoneutral (37°C), HS (42°C)] organoids. Data are shown as the mean±SEM and the differences between means were analyzed using one-way ANOVA followed by Tukey’s multiple comparisons test in biologically independent samples (n = 3–4). Statistical significance between groups was determined at *P* < 0.05. ECM, extracellular matrix; HS, heat stress; 3D, three-dimensional; 2D, two-dimensional.

Oviductal organoids embody miniature self-organized three-dimensional (3D) tissues more faithfully which are accountable in mimicking the key functional, structural, and biological complexity of *in vivo* organs; to date, they have been successfully generated from human (Kessler *et al.*, 2015; Chang *et al.*, 2020; Yucer *et al.*, 2021; Crawford *et al.*, 2024), murine (Xie *et al.*, 2018; Ghosh *et al.*, 2020; Lõhmussaar *et al.*, 2020; Rizo *et al.*, 2025), equine (Thompson *et al.*, 2022; Lawson *et al.*, 2023), feline (Lawson *et al.*, 2023; Thompson *et al.*, 2023), canine (Lawson *et al.*, 2023), porcine (Lawson *et al.*, 2023), bovine (Lawson *et al.*, 2023; Menjivar *et al.*, 2023b), and most recently turkey (Santativongchai *et al.*, 2024) cells. The foregoing paradigm shift in establishing oviductal organoids has enabled their long-term maintenance extending to multiple passages (Kessler *et al.*, 2015; Ghosh *et al.*, 2017; Xie *et al.*, 2018; Lawson *et al.*, 2023), their aptness to retain functional responsiveness under hormonal cues (Crawford *et al.*, 2024), and their ability to be cryopreserved (Thompson *et al.*, 2022, 2023). Accordingly, our overarching aim was to test the hypothesis that oviductal organoids can serve as an *in vitro* surrogate for generating oviductal factors enriched with physiologically relevant oEVs, capable of mitigating the effects of environmental heat stress (HS) on subsequent embryo development and viability. In this regard, the objective of this study was to establish a robust bovine oviductal organoid culture system capable of producing oEVs (Fig. 1A), which may impact the current assisted reproductive technologies for *in vitro* embryo production. Furthermore, a comprehensive and comparative analysis of the EV-miRNA fingerprints of oEVs from our 3D oviductal organoids, 2D monolayer OECs, and *in vivo* OF collections; provided valuable insights into advanced cell culture platforms with the potential for significant clinical implications for improving infertility treatment.

## Materials and methods

### Derivation of bovine oviductal organoids

All experiments involving bovine reproductive material from animal subjects were acquired postmortem from a local abattoir at the discretion of Atlas Meat Company (Fort Collins, CO, USA). Upon collection, intact reproductive tracts (n = 10; a pool of two animals per replicate) were placed on ice until processing at the Animal Reproduction and Biotechnology Laboratory. Reproductive tracts were evaluated for the stage of the estrous cycle, dependent on the presence or absence of a fully formed, active corpus luteum with visible vasculature around its periphery. Resected ovaries containing both complete contralateral and ipsilateral oviducts from assessed stage II diestrus tracts were processed for the generation of oviductal organoids. Excised oviducts were placed in Transport Medium [PBS, 2% anti–anti (Thermo Fisher; Waltham, MA, USA), 100 mg/ml primocin (InvivoGen; San Diego, CA, USA)] within 10 min.

As depicted in the overview represented as the systematic experimental model (Fig. 1A), the isolation of oviductal cells for the establishment of oviductal organoids was performed using previously described methods (Menjivar *et al.*, 2023b). Briefly, ensuing suspensions containing excised oviductal cells were resuspended in 20× (v/v) phenol red-free, growth factor-reduced UltiMatrix (Cultrex; Boitechne BME001-05; Minneapolis, MN, USA) and aliquoted into 25 µl microdrops in a 48-well culture plate (Corning 3548; Corning, NY, USA), and allowed to strengthen formation for 30 min at 37°C. Suspensions were then overlayed with 250 µl of Organoid Medium (OM) DMEM/F-12 without phenol red (Gibco 21041-025; Waltham, MA, USA), 100 μg/ml primocin (Invivogen ant-pm-1), 2.5 mM L-glutamine, 2% B27 Plus (Gibco A35828-01), 1% N2 (Gibco 17502-048), 1% Insulin–transferrin–selenium (Gibco 41400-045), 1 mM nicotinamide (N0636), 50 ng/ml recombinant human EGF (R&D Systems 236-EG; Minneapolis, MN, USA), 50 ng/ml recombinant human FGF-10 (PeproTech 100-26; Waltham, MA, USA), 100 ng/ml recombinant human Noggin (R&D systems 6057-NG/CF), 0.5 μM TGFβ/Alk inhibitor A83-01 (Tocris 2939; Minneapolis, MN, USA), 1.25 mM N-acetyl-L-cysteine (EMD Millipore 106425; Burlington, MA, USA), 10 μM SB202190 (S7067), and 10 μM Y27632 (EMD Millipore 688000) as previously described (Thompson *et al.*, 2020, 2022, 2023). For all experiments, organoid culture suspensions were incubated in a confined chamber regulated to 37°C and 5% CO_2_ in humidified air for growth and development, with OM being renewed by half (v/v) every 2–3 days. At passage 1, Day 7 (14 days from initial collection) (Fig. 1B; Gray Bar), organoids were transferred to VitroGel^®^ (a synthetic, xeno-free extracellular matrix) (TheWell Bioscience VHM04-K; Monmouth Junction, NJ, USA) (Fig. 1B; Green Bar), with the HS group of organoids being transferred 24 h later to a confined chamber regulated to 42°C in an atmosphere of 5% CO_2_ in humidified air (Day 15 from initial culture) for the remaining 24 h culture period (Fig. 1B; Blue Bar). Seeking to offset some of the inherent variability that persists among abattoir-derived reproductive material, the same oviducts were represented in samples subjected to the differing ambient culture conditions (37°C and 42°C) for the purposes of collecting the two subsets of conditioned medium used for the isolation of EVs (N-EVs and S-EVs).

### Enrichment of EVs

For the collection of *in vivo*-derived Ovi-EVs from OF, 2 ml of sterile DPBS (1×) (Thermo Fisher Scientific 14190-144) were flushed through each oviduct of reproductive tracts assessed in the diestrus stage (n = 3 cows; 6 oviducts) using a tuberculin needle and fitted syringe into a 15 ml conical tube for further processing. Additionally, following the collection of conditioned OM (eight replicates/treatment group; ∼10 ml of conditioned OM per replicate), oEVs were enriched using a combinatorial approach (filtration, ultracentrifugation, and size exclusion chromatography), as detailed previously (Menjivar *et al.*, 2023b). Initially, oEV-containing conditioned OM were centrifuged sequentially at 500×*g* for 10 min, 3000×*g* for 10 min, and 17 200×*g* for 30 min and filtered through a 0.22 µm sterile filter (Millex^®^ SLGPR33RB; Waltham, MA, USA), removing residual cellular contaminants, large particles, and protein aggregates. Next, OM were subjected to two consecutive rounds of ultracentrifugation at 120 000×*g* for 70 min in the Optima XE-90 Ultracentrifuge using the SW 55Ti rotor (Beckman Coulter A94471; Brea, CA, USA). All centrifugation steps were performed at 4°C. Enriched oEVs were further standardized by elution in 180 µl of sterile DPBS (1×) (Thermo Fisher Scientific 14190-144) using the Exo-spin™ mini columns (CELL guidance systems EX03-50; Cambridge, UK) according to the manufacturer’s protocol. Enriched oEVs were stored at −80°C for further characterization and molecular analysis.

### Characterization of EVs

Identification and characterization of oEVs were carried out according to predetermined guidelines detailed by the International Society for Extracellular Vesicles (Théry *et al.*, 2018; Welsh *et al.*, 2024), including morphological visualization, nanoparticle concentration/size distribution, and EV marker protein validation. Detailed information regarding experimental procedures has been submitted to the EV-TRACK knowledgebase (https://evtrack.org/) (EVTRACK ID: EV240188) (van Deun *et al.*, 2017).

#### Transmission electron microscopy

**Figure 2.**
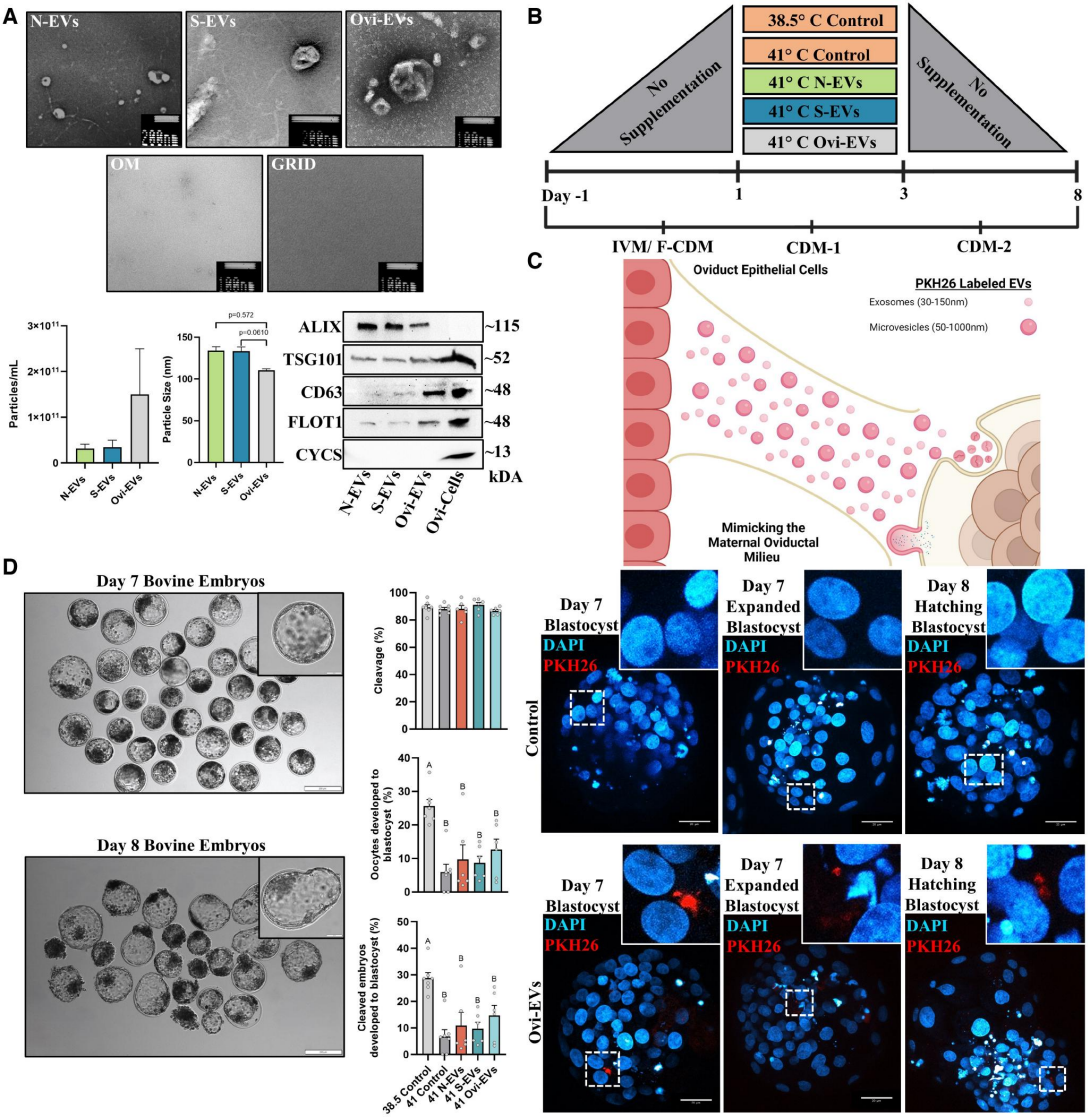
**Oviductal extracellular vesicles (oEVs) exhibit the physiological capacity to augment embryo development under conditions of stress**. (**A**) Identification and characterization of oEVs (Organoids: N-EVs, S-EVs; Oviductal Fluid: Ovi-EVs) by means of transmission electron micrographs indicative of ultrastructural morphology, graphical nanoparticle tracking analysis data representative of sample concentrations (particles per ml) and particle sizes (nm), and western-blotting analysis of persistent and traditionally expressed EV markers (ALIX, TSG101, CD63, FLOT1) and cellular contaminants (CYCS) in enriched EV samples and oviductal cells. (**B**) Schematic representation of the treatment groups, ambient culture environmental temperature applied in CDM-1 during Day 1 to Day 3 of IVC as detailed in Materials and methods section. (**C**) Schematic representation of the superficial oviductal epithelium’s interaction with the developing embryo *in vivo*. Visualization of PKH26-labeled DPBS (1×) Control and Ovi-EVs counterstained with DAPI (pseudo-colored cyan hot) for nuclei visualization in Day 7/8 blastocysts, co-incubated during Day 1 to Day 3 of development. (**D**) Representative Day 7 and Day 8 bovine embryos produced from the addition of oEVs (Organoids: N-EVs, S-EVs; Oviductal Fluid: Ovi-EVs) during CDM-1 from Day 1 to Day 3. Developmental rates (cleavage and 8 dpi blastocyst rates) are presented out of the number of cultured oocytes and cleaved embryos. Data among treatments represent the mean±SEM and the differences between means were analyzed using one-way ANOVA followed by Tukey’s multiple comparisons test from biologically independent rounds (n = 6–8). Statistically significant differences between the treated groups were determined at *P* <0.05. N-EVs, normal organoid EVs; S-EVs, stressed organoid EVs; Ovi-EVs, oviductal fluid EVs; OM, organoid media; Ovi-Cells, oviductal cells; IVM, *in vitro* maturation; CDM, chemically defined medium.

Enriched EVs were imaged by negative staining transmission electron microscopy (TEM) in either the Sperm Morphology and Electron Microscopy Services Laboratory, a clinical and research support service of the Animal Reproduction and Biotechnology Laboratory or through Boulder EM Services Core Facility in the MCDB Department at the University of Colorado, Boulder, CO, USA. EVs were absorbed into mesh copper grids with carbon-coated formvar film (Electron Microscopy Sciences FCF 400-Cu; Hatfield, PA, USA). For this, grids were placed film side down on parafilm containing drops of 1% Alcinan Blue 8GX (Sigma-Aldrich 33864-99-2; St. Louis, MO, USA) for 5 min, followed by washing with ddH_2_O for 15 s. Prior to fixing in 2.4% uranyl acetate (Electron Microscopy Sciences 22400) for 30 s, grids were floated on a 5 µl drop of enriched EVs for 10 min. Grids were imaged on either a JEOL JEM-1200 EX or an FEI/TFS Tecnai T12 Spirit TEM. Processed OM and mesh copper grids without samples were imaged in parallel as internal controls (Fig. 2A). Representative images at various magnifications are provided in Supplementary Fig. S1.

#### Nanoparticle tracking analysis

The particle size distribution and concentrations of EV samples were analyzed using a ZetaView^®^ QUATT 4 Nanosight Instrument (Particle Metrix; Ammersee, Germany). All preparations were performed according to the manufacturer’s instructions. For each analysis, enriched EVs were diluted in sterile DPBS (1×), loaded into the device, and analyzed at 11 positions via scatter mode using a 488 nm laser. Particle size distribution and subsequent concentrations of EV samples were calculated using the Zetaview Software (version 8.05.12 SP1), before and after sample dilution, respectively.

#### Western blotting

Enriched EV samples were suspended in RIPA buffer (Marker Gene™ Technologies M2777; Eugene, OR, USA) and boiled for 5 min with 4× Laemmli Sample Buffer (Bio-Rad 1610747; Hercules, CA, USA) and 2-Mercaptoethanol (Bio-Rad 1610710) at 95°C. Protein lysate samples were separated by SDS polyacrylamide gel electrophoresis (Bio-Rad 4568084) and subsequently transferred to a nitrocellulose membrane (Bio-Rad 1620112). Next, the membrane was blocked at room temperature for 1 h in 5% (w/v) Non-Fat Dry Milk (Bio-Rad 1706404). Subsequently, the membranes were exposed overnight at 4°C with a primary antibody against each marker protein (System Biosciences; rabbit polyclonal ALIX EXOAB-ALIX-1, rabbit polyclonal TSG101 EXOAB-TSG101-1, rabbit polyclonal CD63 EXOAB-CD63A-1, rabbit polyclonal FLOT1 EXOAB-FLOT1-1; Palo Alto, CA, USA) (Sino Biological rabbit polyclonal Cytochrome C 102139-T42; Beijing, China). Following overnight incubation, the membranes were extensively washed with TBS-T solution and exposed at room temperature for 1 h to an appropriate secondary antibody (System Biosciences; Goat anti-Rabbit HRP). After the final steps of repeated washings, SuperSignal™ West Pico PLUS Chemiluminescent Substrate (Thermo Fisher Scientific 34579) was added to the membranes. Imaging was performed using the ChemiDoc XRS+ chemiluminescence system with Image Lab™ Software (version 6.0.1 build 34) (Bio-Rad). Full-length blots are provided in Supplementary Fig. S2.

### Experimental design and *in vitro* embryo production

An overview of the experimental procedures detailing the incorporation of physiologically relevant oEVs in our *in vitro* system for embryo production is detailed in Fig. 2B. Established methods for the production of bovine embryos *in vitro* were adapted from previously published work (Menjivar *et al.*, 2023a). Briefly, bovine ovaries were collected and processed at the ARBL within 2 h of collection. Washed in warmed (37°C) 70% ethanol and repeatedly through physiological saline solution (0.9% NaCl), cumulus–oocyte complexes (COCs) were vacuum-aspirated (CooperSurgical^®^ GPPS-115; Trumbull, CT, USA) from small, growing follicles (3–8 mm in diameter) using an 18-gauge standard hypodermic needle fitted to a 50 ml sterile tube. Viable oocytes surrounded by densely compacted cumulus cells and a visibly homogenous, evenly granulated ooplasm were cultured in groups of 50–75 in 4-well culture dishes (Fisherbrand™ FB012926; Waltham, MA, USA) in 1 ml of CSU chemically defined medium for *in vitro* maturation of oocytes (IVM), pre-equilibrated in a humidified chamber set to 38.5°C in 5% CO_2_ and air.

After 23 ± 1 h of maturation, matured bovine oocytes were transferred to 430 µl of equilibrated CSU chemically defined medium for *in vitro* fertilization (F-CDM/well; Fisherbrand™ FB012926). Frozen-thawed bull spermatozoa were subjected to a multi-layer, 45/90% Percoll^®^ (Sigma-Aldrich P1644) gradient, and centrifuged at 800×*g* for 20 min to separate viable, motile sperm. Following removal of the supernatant, the remaining sperm pellet was washed with 2 ml of CSU chemically defined medium for the handling of early embryos (HCDM-1) and centrifuged at 300×*g* for 5 min. The concentration of spermatozoa was then determined using a hemocytometer, adjusted to 5 × 10^6^ sperm/ml using equilibrated F-CDM, and added to COCs suspended in F-CDM at a concentration of 0.5×10^6^ sperm/ml, and incubated for an additional 18 h in a humidified chamber set to 38.5°C in 5% CO_2_ and air.

The time at which the presumptive zygote would be transiently located within the oviduct *in vivo* occurs from Day 1 until Day 3 (the time in which early embryos develop within Step 1 of our 2-Step culture system in CSU chemically defined medium for *in vitro* culture of early embryos (CDM-1)). Therefore, treatments were arranged for experiments modeling the impact of various oEV-containing mediums on developing embryos exposed to HS as detailed in Fig. 2B. Approximately 18 h later, presumptive zygotes were then vortexed at maximum speed for 60–90 s to slough remaining cumulus cells, transferred through a series of HCDM-1 wash drops, and transferred (in groups of ∼10) to pre-equilibrated micro-droplet culture dishes (Vitrolife 16003 u; Gothenburg, Sweden) containing 25 µl of CDM-1 (oEV-containing micro-drops were adjusted to incorporate ∼1 × 10^8^ total particles) overlayed with ∼4 ml of equilibrated OVOIL (Vitrolife 10029). So far, the physiological concentrations of Ovi-EVs in the oviduct are unknown; however, comparable protein concentrations (0.22–0.42 mg/ml) were used as reference (Almiñana *et al.*, 2017). All treatments were cultured for 24 h in a mixed-gas, humidified chamber regulated to 38.5°C in 5% CO_2_, 5% O_2,_ and 90% N_2_ to stabilize. Following the initial 24 h stabilization period, all treatments aside from the true control (38.5°C Control), were exposed to 41°C for 6 h (adapted from Rivera and Hansen (2001) and tested independently), and returned to homeostatic conditions (38.5°C in 5% CO_2_, 5% O_2,_ and 90% N_2_) for the remainder of the 56 h period. Development was assessed by embryo cleavage in warmed micro-drops of CSU chemically defined medium for the handling of late embryos (HCDM-2), with those embryos containing ≥2 blastomeres then transferred (in groups ≤35 embryos) back to pre-equilibrated 4-well dishes (Fisherbrand™ FB012926) containing 500 µl of CSU chemically defined medium for *in vitro* culture of late embryos (CDM-2) for an ensuing 120 h, in a humidified chamber regulated to 38.5°C in 5% CO_2_, 5% O_2_, and 90% N_2_. Developmental rates were determined by the presence of blastocysts at 7 days post-insemination (dpi) (168 h) and 8 dpi (192 h) and were evaluated according to their respective stage of development and graded morphologically in accordance with the International Embryo Technology Society guidelines (Robertson, 1998).

### Confirmation of EV labeling and uptake

Enriched oEVs from *in vivo*-collected Ovi-Evs, as specified in detail above under ‘Enrichment of EVs’, were labeled with PKH26 (Sigma-Aldrich Mini26-1KT), a red fluorescent linker dye responsible for labeling lipid membranes as preciously described (Menjivar *et al.*, 2023a). Briefly, oEVs (2.5 × 10^11^ particles/ml) or DPBS (1×; Control) were incubated with 1 µl of PKH26 (4 × 10^−6^ M) for 5 min at room temperature in the dark, followed by immediate quenching with 1% BSA (Sigma-Aldrich A6003) for 1 min. Samples were precipitated using the Exo-spin™ buffer-exosome precipitation reagent (CellGS^®^ EX06-30) for 60 min in the dark at 4°C, then centrifuged at 16 000×*g* for 30 min and resuspended in 100 μl of DPBS (1×) before purification using the Exo-spin™ columns according to the manufacturer’s protocol.

Recently, oEVs from OECs incorporation into 8-cell stage embryos *in vivo* has been evidenced in a murine model (Stephens *et al.*, 2024). Therefore, our aim was to investigate whether or not the application and uptake of Ovi-EVs could be evidenced in fully developed embryos. At Day 1, groups of ∼10 presumptive zygotes were transferred to pre-equilibrated micro-droplet culture dishes (Vitrolife 16003 u) containing 25 µl of CDM-1 (containing ∼1 × 10^8^ total fluorescently labeled particles) overlayed with ∼4 ml of equilibrated OVOIL (Vitrolife 10029) and cultured for 56 h in a mixed-gas, humidified chamber regulated to 38.5°C in 5% CO_2_, 5% O_2,_ and 90% N_2_. Cleaved embryos were then transferred to pre-equilibrated CDM-2 as detailed previously, and at 7/8 dpi, developed embryos were fixed in 4% paraformaldehyde (Electron Microscopy Services 15710), washed in DPBS (1×)-PVP (0.1%) (Sigma-Aldrich P0930) and assessed for the internalization of oEVs using an inverted laser scanning confocal microscope (Carl Zeiss Microscopy ZEISS LSM980; Oberkochen, Germany).

### RNA isolation and qRT-PCR analyses

Total RNA was isolated from organoids (3–4 replicates/treatment group, a pool of samples from two animals per replicate) using the RNeasy^®^ Plus Micro Kit (Qiagen 74034; Hilden, Germany). Alternatively, total RNA was isolated from single whole embryos (n = 108 total embryos) using the Arcturus™ PicoPure™ RNA Isolation Kit (Thermo Fisher Scientific KIT0204). During the extraction, removal of genomic DNA contaminants was performed via on-column DNA digestion using RNase-free DNase (Qiagen 79254). Total RNA from organoid and embryo samples was reverse transcribed in a total reaction volume of 20 µl using the SuperScript™ III First-Strand Synthesis Super Mix Kit (Invitrogen 18080400; Waltham, MA, USA). Real-time PCR was performed using a CFX96 Touch Real-Time PCR Detection System (Bio-Rad) with iQ SYBR Green Supermix (Bio-Rad 1708880) using Integrated DNA Technologies primers (Supplementary Table S1) and previously described methods (Menjivar *et al.*, 2023b). In all cases, *GAPDH* and *β-Actin* were used as reference genes.

### Histology, immunohistochemistry, and immunofluorescence

Immunolocalization studies were in part performed using previously described methods (Menjivar *et al.*, 2023b). Briefly, organoids were fixed in 4% paraformaldehyde (Electron Microscopy Services 15710) for 30 min and embedded in 2% agarose gel (Bio-Rad 1613101), dehydrated in ethanol, embedded in paraffin wax, and sectioned (6 μm)/stained using hematoxylin and eosin (H&E). Sections were then mounted on slides, deparaffinized, and rehydrated through a series of graded alcohols until distilled water. Deparaffinized sections were then subjected to antigen retrieval by incubation in citrate buffer (pH 6; 96°C for 20 min), blocked with hydrogen peroxide, washed in distilled water, and left to incubate with primary antibodies overnight at 4°C. The slides were washed and incubated with HRP-conjugated and/or biotinylated secondary antibodies (OVGP1; Biotin Ms IgG, ZO1; Rb IgG HRP, and FOXJ1; Biotin Rb IgG), respectively. Immunohistochemical staining was activated using the RTU Vectastain kit (Vector Laboratories; Newark, CA, USA) for 30 min and visualized using Vectastain ABC-HRP and DAB substrate solution (Vector Laboratories), lightly counterstained with hematoxylin prior to affixation with a coverslip.

Alongside this, whole, intact organoids and fully developed embryos were fixed for 20 min in 4% paraformaldehyde (Electron Microscopy Services 15710) and then stored in DPBS (1×) (Thermo Fisher Scientific 14190-144) with 0.5% Bovine Serum Albumin (Sigma A6003) until staining. Samples were washed three times in DPBS (1×) (Thermo Fisher Scientific 14190-144) with 0.1% polyvinylpyrrolidone (PVP; Sigma P0930) and 0.1% Tween20 (Bio-Rad 1706531) and then permeabilized in DPBS (1×) (Thermo Fisher Scientific 14190-144) with 0.3% Triton™ X-100 (TX100; Sigma X100) (30 min). After blocking (2 h) in DPBS (1×) (Thermo Fisher Scientific 14190-144) with 0.1% Tween20 (Bio-Rad 1706531), 1.0% Bovine Serum Albumin (Sigma A6003), 0.1 M Glycine (Sigma G7126) and 10% (v/v) horse serum, samples were then washed and incubated with primary antibodies (at minimum 24 h, 4°C). Following three washes in DPBS (1×) (Thermo Fisher Scientific 14190-144) with 0.1% polyvinylpyrrolidone (PVP; Sigma P0930) and 0.1% Tween20 (Bio-Rad 1706531), samples were incubated (1 h) with the appropriate secondary antibodies. A detailed list of the antibodies is provided in Supplementary Table S2. Samples were counterstained with DAPI (500 nM; Invitrogen D1306) in solution (DPBS (1×) (Thermo Fisher Scientific 14190-144) with 0.1% polyvinylpyrrolidone (PVP; Sigma P0930)) (20 min). Fluorescent signals were visualized using an inverted laser scanning confocal microscope (Carl Zeiss Microscopy ZEISS LSM980) with images acquired as z-projections and czi. projects analyzed using ImageJ software (National Institute of Health; Bethesda, MD, USA).

### Data mining and bioinformatic analyses of oEVs miRNA cargo

Data were obtained from studies involving 3D, 2D, and *in vivo* collected oEVs of oviduct origin profiled for their miRNA cargoes. Our previously generated study was used (Menjivar *et al.*, 2023b) along with publicly available data (Hamdi *et al.*, 2021; Mazzarella *et al.*, 2021), data downloaded from the data set GSE110443 (Almiñana *et al.*, 2018) in the Gene Expression Omnibus, data downloaded from the data set PRJNA928588 (Hamdi *et al.*, 2024) in the Sequence Read Archive, and/or data obtained via request from the authors (Leal *et al.*, 2022; Reshi *et al.*, 2023), as indicated in Table 1. Only those miRNAs relevant to the early luteal phase were included within the comparison. For studies reporting stage-specific data, we selectively utilized miRNAs expressed during the early luteal phase, while for studies reporting only the commonly expressed miRNAs spanning various stages, only those miRNAs that were mutually expressed during that of those in the early luteal phase were used for subsequent analysis.

**Table 1.** Overview of the data sets employed for data analysis.

Accession no.	Main characteristics	Total n	References
GSE110443^a^	*In vivo*-collected EVs from bovine oviductal fluid at different stages of the estrous cycle.	20 (5 replicates, 4 stages)	Almiñana *et al.* (2018)
^b^	*In vivo*-collected EVs from the isthmus of ipsilateral oviducts from pregnant and non-pregnant cows.	12 (6 replicates, 2 groups)	Mazzarella *et al.* (2021)
^b^	*In vivo*-collected EVs from bovine oviduct and uterine fluid across different stages of the estrous cycle.	24 (3 replicates, 2 groups/ 4 stages)	Hamdi *et al.* (2021)
^b^ ^,^ ^c^	*In vivo*-collected EVs from bovine oviduct and uterine fluid used sequentially *in vitro*, improve bovine embryo quality.	6 (3 replicates, 2 groups)	Leal *et al.* (2022)
^c^	*In vitro*-produced EVs from 2D bovine oviductal epithelial cells contain altered EV cargo in response to spermatozoa.	9 (3 replicates, 3 groups)	Reshi *et al.* (2023)
GSE221895^a^	*In vitro*-produced EVs from 3D bovine oviductal organoids subjected to thermoneutral and heat stress conditions.	6 (3 replicates, 2 groups)	Menjivar *et al.* (2023b)
PRJNA928588^d^	*In vitro*-produced EVs from 2D BOECs in response to embryos of diverging quality.	18 (3 replicates, 6 groups)	Hamdi *et al.* (2024)

Data sets were re-analyzed and cohesively integrated to identify similarities in EV-miRNA expression patterns from *in vitro*- (2D and 3D) and *in vivo*-collected EVs of oviduct origin.

a Accession numbers correspond to NCBI’s Gene Expression Omnibus (GEO) database.

b Study employed quantitative real-time PCR to determine the relative abundance of known mature miRNAs following the protocol of Da Silveira *et al.* (2018).

c Requested from authors.

d Accession numbers correspond to NCBI’s Sequence Read Archive (SRA) database.

Data were processed and the normalized values (CPM) of the expressed miRNAs from the small RNAseq studies (Almiñana *et al.*, 2018; Menjivar *et al.*, 2023b; Reshi *et al.*, 2023; Hamdi *et al.*, 2024) were used to generate the principal component analysis (PCA) using Past 4.17c software (Hammer *et al.*, 2001) and the heatmap using the heatmap.2 (gplots) in R. The intersections of the expressed miRNAs among the different studies were visualized using the UpSet R package (Lex *et al.*, 2014; Conway *et al.*, 2017). Based on the intersections between all of the incorporated studies, the group of 88 miRNAs commonly expressed between our previously published 3D organoid oEVs and at least two of the incorporated *in vivo* studies (albeit not those expressed among the *in vitro* studies) were extracted for further analysis. Among this group of 88 miRNAs, five (bta-miR-148a, bta-miR-122, bta-miR-30d, bta-miR-10b, bta-miR-192) were among the list of the top 20 expressed miRNAs in our 3D study involving oEVs secreted from organoids. Genes targeted by the five commonly expressed miRNAs were identified using the TargetScan analysis tool (Agarwal *et al.*, 2015) with a threshold of cumulative weighted context ++ score ≤ −0.3. The list of predicted target genes was submitted to the DAVID bioinformatics web tool (https://david.abcc.ncifc rf.gov/) for ontological classification. Significant pathways were identified from the Kyoto Encyclopedia of Genes and Genomes (KEGG) database (Kanehisa and Goto, 2000). Interaction networks of the targeted genes and the identified pathways were constructed by Cytoscape (https://www.cytoscape.org/).

### Statistical analysis

All quantitative data were analyzed in GraphPad Prism 10 (GraphPad; San Diego, CA, USA). Gene expression analyses are shown as the mean±SEM and the differences between means were analyzed using one-way ANOVA followed by Tukey’s multiple comparisons test in biologically independent samples. Statistical significance between groups was determined at *P* < 0.05.

## Results

### Development of biomimetic bovine oviductal organoids

The bovine oviducts, luminally lined by intricately folded pseudostratified epithelial cells, were excised from diestrus reproductive tracts, isolated using enzymatic digestion, and cultured in a basement membrane extract (BME) under defined conditions (Fig. 1A) (Menjivar *et al.*, 2023b). During the 16-day (Passage 1 Day 9; P1D9) experiment (Fig. 1B), those cells rapidly self-organized into organoid-like structures (with apical-in polarity by histological morphology) that grew substantially larger over time. After ∼7 days (P1) of culture, the round, cystic organoid structures were dissociated to predominantly single cell/cell clusters that would self-initiate the regeneration of proliferative organoids. In our previous study, we observed oviductal organoid retention of the *in vivo* oviduct (Naïve OECs; Menjivar *et al.*, 2023b), as well as their aptness to respond to exogenous steroid hormone stimulation following cryopreservation and long-term culture (P2D8; Control, Estrus, Diestrus) (unpublished data).

Mature organoids withdrawn from BME encapsulation (>14 days) morphologically constituted 3D structures, evidenced by the double-stranded DNA staining of DAPI among the 2D cross-sections of the 3D projections of intact organoids. Immunofluorescence analysis revealed the hallmark capacity of oviductal organoids to stably express ZO1 (functional tight junctions amid the apical membrane of epithelium), AC-Tub (hair-like, acetylated microtubules of ciliated cells), and PAX8 (localized secretory cell marker of healthy OECs) (Fig. 1C). With respect to the similarity of organoid cell-network architectures to that observed *in vivo*, cross-sectional staining of H&E, OVGP1, ZO1, and FOXJ1, confirmed that oviductal organoids were composed of a monolayer of polarized columnar epithelial cells (Fig. 1D), a simplified model similar to that of the complex hierarchy visualized in native tissues (Menjivar *et al.*, 2023b). To gauge the impact of our HS model, we then performed real-time quantitative PCR assays (Fig. 1E; Supplementary Fig. S3) measuring a gene panel consisting of genes involving prostaglandin synthesis (*PTGS2*, *PTGES*), hormone receptors (*PGR*, *ESR1*, *OXTR*), cell proliferation and adhesion (*MKI67*, *CDH1*), tight junctions (*OCLN*, *TJP1*), oviduct-specific glycoprotein (*OVGP1*), and ciliated cell function and differentiation (*LRRC6*, *TUBA1A*, *TUBA1B*, *TUBA1C*, *FOXJ1*). Following one to two passages, discernible differences persisted in the extent to which patterns of genes were expressed among non-hormone-stimulated organoids extending various passages (Control; P2D8) (37°C, 42°C; P1D9). For example, *OCLN* and *TJP1*, demarcating markers of functional tight junctions, were significantly increased in 37°C (*P* = 0.0155 and *P* = 0.0159, respectively) and 42°C (*P* = 0.0155 and *P* = 0.0019, respectively) organoids compared to the Control. Moreover, the retention in our model of an *in vivo* signature was in part further corroborated through *TUBA1A*, *TUBA1B*, and *TUBA1C*, alpha-tubulin isoforms involved in the formation of microtubules, that were significantly increased in 37°C (*P* = 0.0017, *P* = 0.1820, and *P* = 0.0004, respectively) and 42°C (*P* = 0.0004, *P* = 0.0170, *P* < 0.0001, respectively) organoids compared to the Control.

### EVs of oviductal origin functionally regulate embryo development

Collection and characterization of oEVs from *in vitro*-cultured oviductal organoids [N-EVs (37°C) and S-EVs (42°C)] and *in vivo*-collected OF (Ovi-EVs) were initially subjected to a combinatorial enrichment approach (filtration, ultracentrifugation, and size exclusion chromatography). Representative TEM images of encapsulated EV-like particles from each of the subsequent treatment groups are shown in Fig. 2A and Supplementary Fig. S1. When comparing *in vitro*-derived (N-EVs, S-EVs) with *in vivo*-collected (Ovi-EVs) oEVs, a higher number of total particles per ml was recovered *in vivo* (1.5 × 10^11^ particles per ml; 3.15 × 10^10^ and 3.49 × 10^10^, N-EVs and S-EVs, respectively), with a trend of smaller particles (110.25 nm) compared to the average diameter of those recovered from 3D organoids *in vitro* (133.55 nm). Western blotting (WB) analyses confirming the presence of EV protein markers (ALIX, TSG101, CD63, FLOT1) and void of cellular contaminants (CYCS) were also performed in oEVs retrieved from conditioned organoid media (OM) and OF, as well as in oviductal cells, an indication of the sample yields and purity (Supplementary Fig. S2).

Under the physiologically relevant time point in which the transient presumptive zygote would presumably progress through the oviduct *in vivo*, the *in vitro* medium compositions were preset (Day 1–Day 3) to mimic the maternal signals shuttled to developing embryos under conditions of HS (41°C) (Fig. 2B). Mimicking maternal signals (oEVs) absent in the current *in vitro* system, we then aimed to confirm their ability to permeate and pass through the zona pellucida for internalization in bovine embryos. Notably, the addition of labeled oEVs were stably visible in fully developed Day 7 and Day 8 blastocysts derived from the addition of oEVs on Day 1 in the form of punctate and perinuclear structures, suggesting that endocytosis spontaneously occurs in the absence of catalyzing reagents (Fig. 2C). To further determine the effect of N-EVs, S-EVs, and Ovi-EVs on embryo development under conditions of regulated ambient HS, cycles were carried out until Day 8, thus allowing any carryover influence to be quantified. While cleavage rates were not majorly affected by the addition of oEVs aside from their origin or the regulated application of ambient HS, the blastocyst per oocyte (41°C Control: *P* = 0.0001; 41°C N-EVs: *P* = 0.0035; 41°C S-EVs: *P* = 0.0017; 41°C Ovi-EVs: *P* = 0.0250) and blastocyst per cleaved embryo (41°C Control: *P* = 0.0003; 41°C N-EVs: *P* = 0.0062; 41°C S-EVs: *P* = 0.0031; 41°C Ovi-EVs: *P* = 0.0484) rates were significantly impacted by HS compared to the 38.5°C Control (Fig. 2D).

### Improved embryo quality is associated with oEV internalization in bovine preimplantation embryos

**Figure 3.**
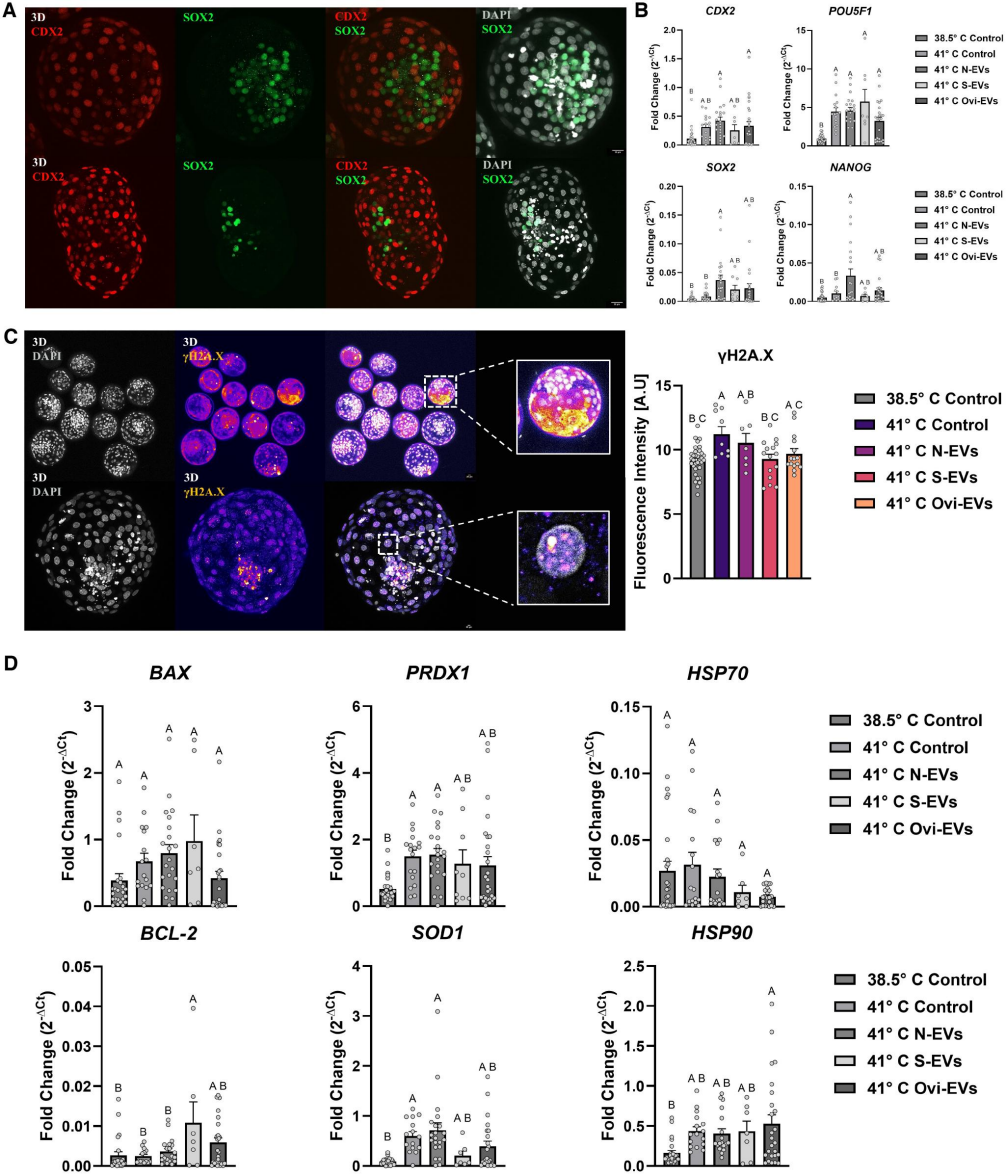
**Effects of culture environment and oviductal extracellular vesicles (oEVs) on safeguarding the quality of developing embryos**. (**A**) 3D representations of bovine embryos (expanded and hatching) stained for CDX2 (red) and SOX2 (green) and counterstained with DAPI (pseudo-colored gray) for nuclei visualization (Scale bar, 20 µm). (**B**) Individual blastocyst gene expression for pluripotency and embryo quality markers (*POU5F1*, *NANOG*, *SOX2*, *CDX2*) (n = 108). (**C**) 3D representations and fluorescence quantification of group and individual bovine embryos stained for phospho-γH2A.X (pseudo-colored fire) and DAPI (pseudo-colored gray) for nuclei visualization (n = 81; Scale bar, 20 µm). (**D**) Individual blastocyst gene expression for apoptosis (*BAX*, *BCL2*), oxidative stress (*PRDX1*, *SOD1*), and heat stress (*HSP70*, *HSP90*) markers (n = 108). Data are shown as the mean±SEM and the differences between means were analyzed using one-way ANOVA followed by Tukey’s multiple comparisons test. Statistically significant differences between the treated groups and the control were determined at *P* < 0.05. oEVs, oviductal EVs; N-EVs, normal organoid EVs; S-EVs, stressed organoid EVs; Ovi-EVs, oviductal fluid EVs; 3D, three-dimensional.

Next, we performed co-immunostaining for trophectoderm (TE; CDX2) and inner cell mass (ICM; SOX2) markers which revealed that using 3D reconstruction and overlay imaging techniques, SOX2-positive cells were localized exclusively within the ICM, whereas CDX2-positive cells were segmented amidst the outer layer of TE cells in both expanded and hatching Day 7 blastocysts (Fig. 3A). Although drastic changes among individual preimplantation embryos did not persist among traditional pluripotency markers (*CDX2*, *SOX2, POU5F1*, *NANOG)*, oEV supplementation (N-EVs, S-EVs, Ovi-EVs) under conditions of HS compared to the non-supplemented 38.5°C and 41°C Control appeared to be variably affected. For instance, compared to the 38.5°C and 41°C Controls, *NANOG* (*P* = 0.0004, *P* = 0.0489, respectively) and *SOX2* (*P* = 0.0007, *P* = 0.0260, respectively) expression levels were significantly altered in embryos derived from N-EVs supplementation, whereas CDX2 expression was altered predominantly among embryos derived from the co-incubation of both N-EVs (*P* = 0.0024) and Ovi-EVs (*P* = 0.0418) as compared only to the 38.5°C Control. Additionally, although *POU5F1* was significantly altered among embryos derived from treatments containing oEVs (N-EVs: *P* < 0.0001; S-EVs: *P* < 0.0001; Ovi-EVs: *P* = 0.0045) when compared to the 38.5°C Control, no discernible differences persisted with regard to the 41°C Control (Fig. 3B). Consistent with these results, we observed fluorescent mark γH2A.X accumulation in bovine embryos, indicative of a response in collated DNA damage. The results indicate that specifically under conditions of HS, preimplantation embryos that are primed with S-EVs from oviductal organoids exposed to elevated ambient temperatures have significantly less accumulated DNA damage in fully developed blastocysts (*P* = 0.0142) compared to embryos derived from the non-supplemented 41°C Control, suggestive of the capacity of S-EVs as a safeguarding mechanism for developing embryos under conditions of stress to mitigate total DNA damage to levels of the non-stressed counterparts (38.5°C Control) (Fig. 3C).

Next, although differences in the expression patterns from individual embryos did not persist in levels of pro-apoptotic factor *BAX*, embryos derived from the addition of S-EVs from oviductal organoids exposed to elevated ambient temperatures contained increased levels of anti-apoptotic factor *BCL-2* in fully developed blastocysts compared to both the non-supplemented 38.5°C and 41°C Controls (*P* = 0.0093 and *P* = 0.0154, respectively), suggestive of their protective potential. Under subsequent conditions of HS, levels of beneficial antioxidant enzymes encoded by genes *PRDX1* and *SOD1* significantly increased from N-EV supplementation (*P* = 0.0096 and *P* < 0.0001, respectively) compared to those embryos from the non-supplemented 38.5°C Control, albeit not significantly altered when compared to the 41°C Control. Additionally, although not statistically significant, among embryos derived from the addition of oEVs, a lowered expression of *HSP70* trended below the non-supplemented 38.5°C and 41°C Controls, indicative of a potential functional mitigation strategy to applied HS by reducing its severity. Notably and inversely connected to *HSP70*, comparing embryos derived from the non-supplemented 41°C Control and those resulting from the addition of oEVs, the expression levels of *HSP90* were not conclusively affected, characteristic of their potential role in a partly counterbalance effort, without the complete neutralization of the negative effects of HS insults on developing embryos (Fig. 3D).

### Embryonic differences in DNA methylation and histone modifications drive perturbations in transcriptional regulation

**Figure 4.**
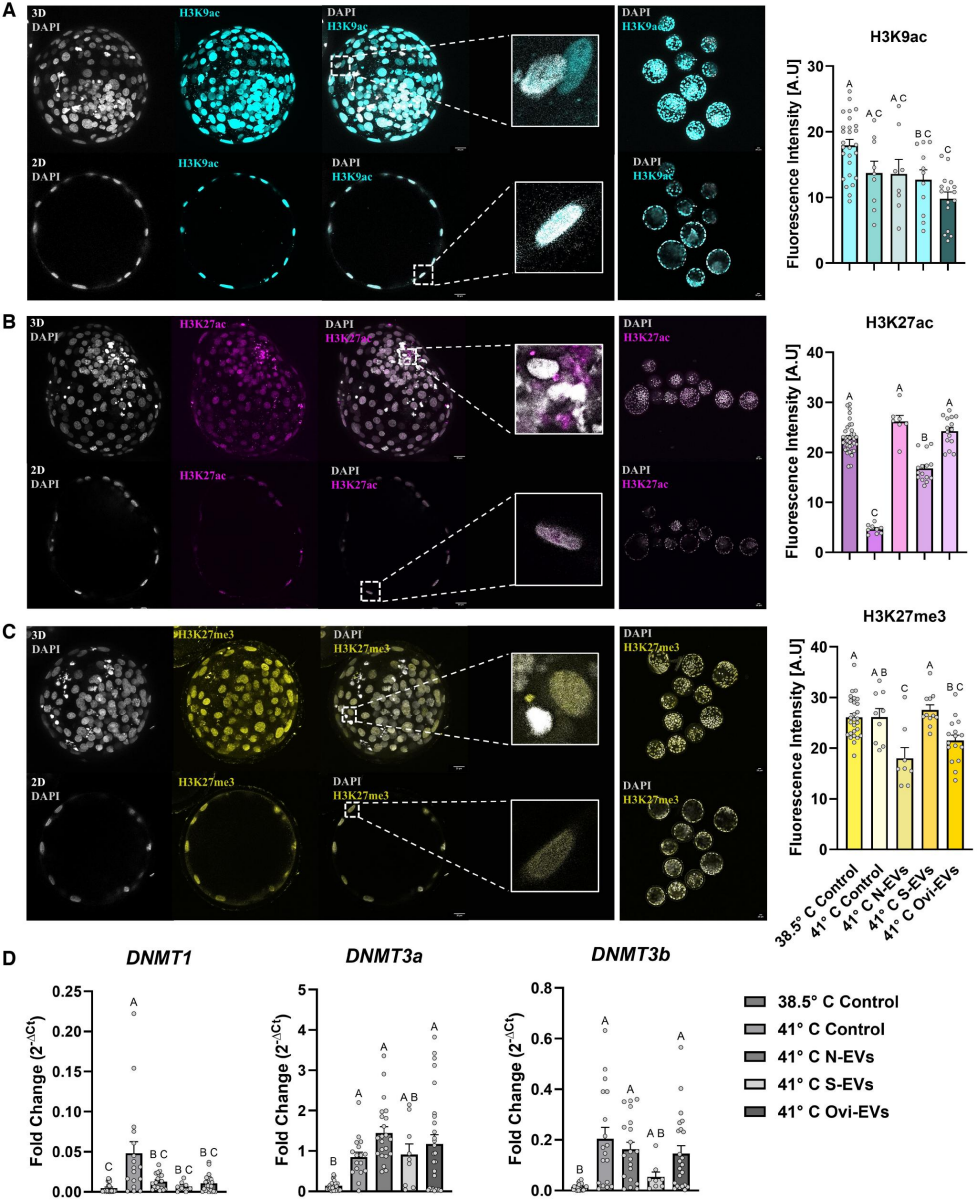
**Epigenetic reprogramming of bovine embryos is associated with the addition of oviductal extracellular vesicles (oEVs) during defined heat stress exposure on subsequent development**. 3D/2D representations and fluorescence quantification of individual and group bovine embryos stained for H3K9ac (**A**) (pseudo-colored cyan) (n = 71), H3K27ac (**B**) (pseudo-colored magenta) (n = 81), and H3K27me3 (**C**) (pseudo-colored yellow) (n = 71), all counterstained with DAPI (pseudo-colored gray) for nuclei visualization (Scale bar, 20 µm). (**D**) Individual blastocyst gene expression for dynamic epigenetic reprogramming of DNA marks (*DNMT1*, *DNMT3A*, *DNMT3B*) (n = 108). Data are shown as the mean±SEM and the differences between means were analyzed using one-way ANOVA followed by Tukey’s multiple comparisons test. Statistically significant differences between the treated groups and the control were determined at *P* < 0.05. oEVs, oviductal EVs; HS, heat stress; N-EVs, normal organoid EVs; S-EVs, stressed organoid EVs; Ovi-EVs, oviductal fluid EVs; 3D, three-dimensional; 2D, two-dimensional.

To determine the impact of oEVs (N-EVs, S-EVs, Ovi-EVs) on their potential influence to the epigenetic landscapes of developing embryos due to HS, we observed their perturbations to H3K9ac, and competitive marks H3K27ac and H3K27me3, in association to their relative expressions of hallmark DNA methyltransferases (*DNMT1*, *DNMT3A*, *DNMT3B*) among individual embryos of undisclosed sexual phenotypes. From the immunolabeled 3D reconstruction and 2D cross-section images, preferential segmentation into TE and ICM cells was not evaluated; thus, images were analyzed as reconstructed whole embryos. This analysis revealed the marked tendency of oEV supplementation regardless of their origin (*in vitro* vs *in vivo*) to attenuate fluorescent mark H3K9ac among developed blastocysts compared to those derived from their non-treated 38.5°C Control counterparts (S-EVs: *P* = 0.0394, Ovi-EVs: *P* < 0.0001) (Fig. 4A).

Next, competitive bivalent marks H3K27ac and H3K27me3 were evaluated to determine the influence of oEVs (N-EVs, S-EVs, Ovi-EVs) on the established compensatory notion that the loss of H3K27me3 and subsequent gain of H3K27ac precede transcriptional activation (Pasini *et al.*, 2010). Immunofluorescence analyses of blastocysts derived from the non-supplemented 38.5°C and 41°C Control groups exhibited a pronounced heat exposure-dependent decline in H3K27ac (*P* < 0.0001). Notably, blastocysts derived from HS exposure in the presence of the addition of oEVs (N-EVs: *P* < 0.0001; S-EVs: *P* < 0.0001; Ovi-EVs: *P* < 0.0001) showed the contrary, with significant increases in epigenetic mark H3K27ac to basal thermoneutral levels (38.5°C Control), compared to those from the non-supplemented 41°C Control (Fig. 4B). Conversely and in competitive nature, a marked trend for the reduction of H3K27me3 persists with blastocysts derived from oEV supplementation (N-EVs and Ovi-EVs) aside from their origin (*in vitro* vs *in vivo*) compared to the controls under both HS and thermoneutral conditions (N-EVs: *P* = 0.0055 and *P* = 0.0005, respectively; Ovi-EVs: *P* = 0.1523 and *P* = 0.0245, respectively) (Fig. 4C). Additionally, we found that oEVs attenuate the global expression of *DNMT1*, to levels similar of embryos derived from thermoneutral conditions (38.5°C Control), compared to embryos derived under condition of HS (41°C Control) (N-EVs: *P* = 0.0048; S-EVs: *P* = 0.0112; Ovi-EVs: *P* = 0.0008) (Fig. 4D).

### 3D cellular architecture favors microRNA crossover with *in vivo* bovine oEVs

**Figure 5.**
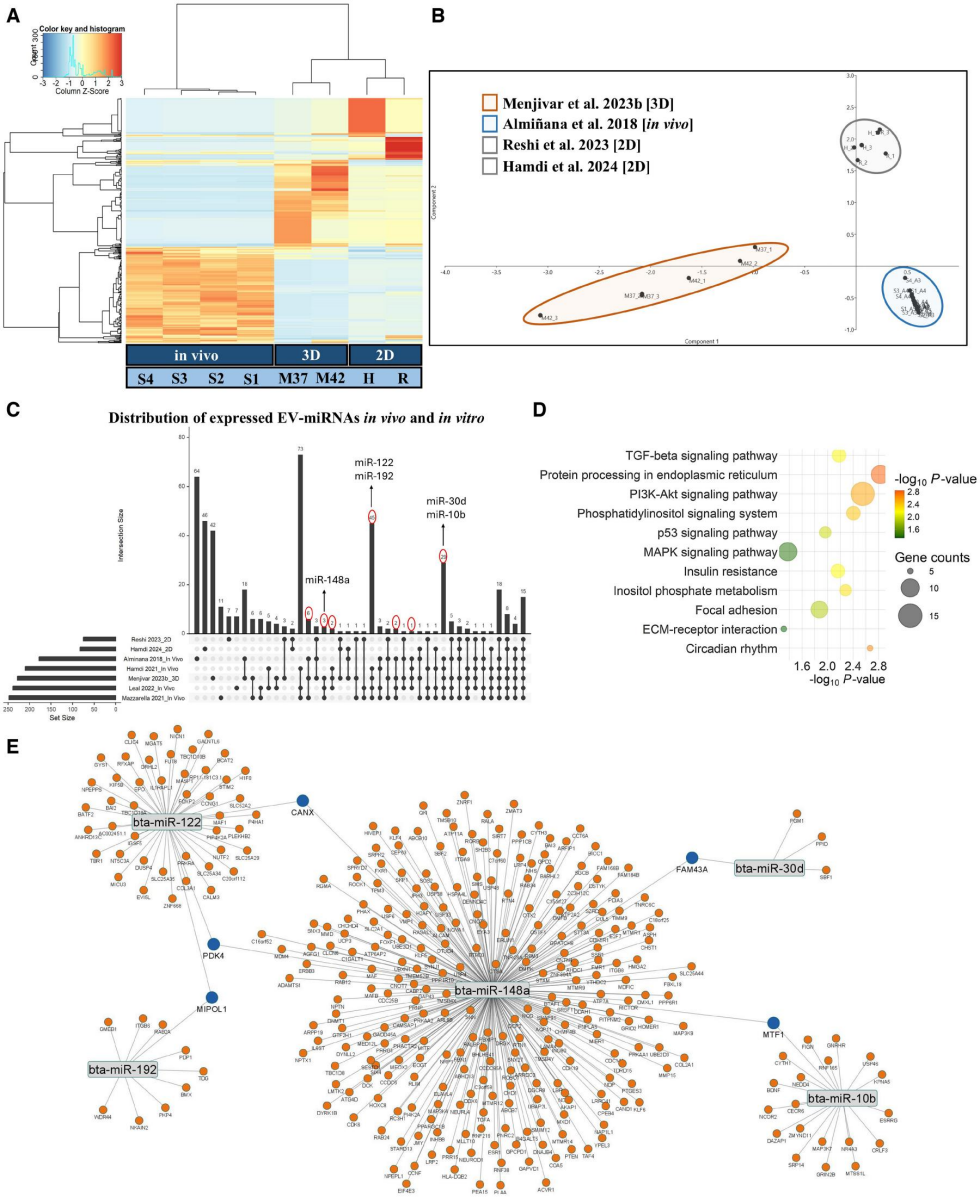
**Integration and comparative meta-analysis of extracellular vesicle-derived miRNA (2D, 3D, *in vivo*) profiles in oviductal extracellular vesicles (oEVs)**. (**A**) Heatmap illustrating the similarity in miRNA profiles between *in vivo* and 2D monolayer OEC datasets, and integrated 3D organoids, with color intensity representing the column z-score. (**B**) Two-dimensional principal component analysis (PCA) representation of the clustered groups [(2D; gray) (3D; orange) (*in vivo*; Blue)] based on the expressed EV-miRNAs as detailed in the Materials and methods section. (**C**) UpSet plot showing the intersection size of the individual datasets represented in the lower left corner of the image, with a detailed description of each study in Table 1. Specific datasets involved in each intersection are identified with connected solid black circles under the vertical bars, with unconnected circles representing pairs that are found exclusively in the corresponding datasets. Among the detailed miRNAs are those cross-referenced from the list of expressed miRNAs found in our previously published and integrated 3D organoid oEVs and the *in vivo* oEVs, which were among the top 20 highly expressed. (**D**) Ontological classification signified by the bubble plots reveals the top 11 significant pathways and biological processes targeted by the five miRNAs most highly expressed in our 3D organoid oEVs study, with the color and size of each bubble representing the *P*-value and number of miRNA target genes, respectively. (**E**) Interactive network of the five miRNAs (represented in gray) most highly expressed in 3D organoids and their experimentally validated target genes (represented in orange, with mutual targets identified in blue). oEVs, oviductal EVs; S, Alimiñana *et al*. (2018); M, Menjivar *et al.* (2023b); H, Hamdi *et al.* (2024); R, Reshi *et al.* (2023).

EVs derived from bovine oviductal organoids exposed to thermoneutral and HS conditions (N-EVs and S-EVs) were collected, and their miRNA profiles were reported in our previously published dataset (Menjivar *et al.*, 2023b). Incorporating the studies listed in Table 1 allowed for an *in silico* comparison of the miRNAs detected in oEVs from *in vivo* (Almiñana *et al.*, 2018; Hamdi *et al.*, 2021; Mazzarella *et al.*, 2021; Leal *et al.*, 2022), 2D OECs (Reshi *et al.*, 2023; Hamdi *et al.*, 2024), and 3D oviductal organoid (Menjivar *et al.*, 2023b) origins. Notably, through the hierarchical heatmap and 2D PCA plot, after 16 days of culture, organoids were shown to secrete oEVs with an intermediary miRNA profile compared to that of those found *in vivo* and those of 2D OECs (Fig. 5A and B). Visualized in the UpSet plot (Fig. 5C), the intersection of the datasets (*in vivo*, 2D, 3D) incorporated for the meta-analysis showed the detection of 456 miRNAs among oEVs. In particular, 55 miRNAs were found exclusively in the 2D oEVs, 42 miRNAs exclusively in the 3D oEVs, and 169 miRNAs were exclusive to the oEVs collected from *in vivo* OF. Comparatively, 4 miRNAs were found to be exclusively expressed among the 2D and 3D studies, 4 miRNAs exclusive among the 2D and *in vivo* studies, and 116 miRNAs were exclusive in the 3D and *in vivo* studies (Supplementary Table S3).

We then mechanistically performed target gene prediction analysis for the identification of key validated genes and/or pathways regulated by the subset of miRNAs exclusive to the oEVs in at least two *in vivo* studies and among the top 20 highly expressed miRNAs list from that of the 3D study (identified in Fig. 5C) and their potential involvement in the regulation of embryonic development. Pathway analysis revealed that genes targeted by EV-miRNAs found exclusively among the 3D and *in vivo* studies were involved in TGF-beta, PI3K-Akt, p53, and MAPK signaling, alongside inositol phosphate metabolism and circadian rhythm (Fig. 5D). Furthermore, interaction networks between common experimentally validated target genes of the five EV-miRNAs exclusive to the 3D and *in vivo* studies are presented in Fig. 5E.

## Discussion

Organoids of oviduct origin provide fitter tissue modeling systems than monolayer cultures (Kessler *et al.*, 2015; Xie *et al.*, 2018; Chang *et al.*, 2020; Ghosh *et al.*, 2020; Lõhmussaar *et al.*, 2020; Thompson *et al.*, 2022, 2023; Lawson *et al.*, 2023; Menjivar *et al.*, 2023b), particularly as an acceptable platform for better understanding preimplantation embryo–maternal crosstalk at the molecular magnitude amid the largely inaccessible tubal conduit (Haider and Beristain, 2023). In this study, we developed an uncharted approach using a simple methodology for the fabrication of bovine oviductal organoids cultured in a BME substrate, enabling the production and subsequent collection of physiologically pertinent oEVs carrying molecular cargos functionally capable of augmenting the development of *in vitro*-cultured embryos. As the transient presumptive zygote migrates through the oviduct *in vivo*, we anticipate that the generation of apical-in (AI) organoids will facilitate proper acceptance as a suitable model for understanding the maternal physiology under conditions of applied stress and for the collection of maternal signals (oEVs) to reckon its absence in the current *in vitro* system, as has occurred for polarity reversed endometrial models recapitulating attachment and implantation events (Rawlings *et al.*, 2021; Ahmad *et al.*, 2024; Saadeldin *et al.*, 2024; Shibata *et al.*, 2024). We show that bovine oviductal organoids effectively mimic several aspects of the *in vivo* oviduct by: (i) organoids propagate differentiated cell types, stably express critical genes with pertinent functional governance, and express principal proteins/model the spatial positioning of cells that line the bovine oviduct (Fig. 1); (ii) oEVs secreted from 3D organoid cultures functionally mediate embryonic crosstalk, safeguarding and reinforcing embryo quality (Figs 2, 3, and 4); and harbor exclusively expressed miRNAs perplexed with oEVs from *in vivo* OF (Fig. 5), that are likely in part responsible for moderating some of the key functional characteristics of the developing preimplantation embryo.

Important developmental milestones achieved *ex vivo* highlight the ability of organoids to resemble the architecture, structure, and function of their native tissue, recapitulating key features such as tissue regeneration and homeostasis. Organoids cultured in BME substrate, independent of hormone stimulation, stably expressed genes related to prostaglandin function (*PTGS2*, *PTGES*) (Gauvreau *et al.*, 2010), hormone receptors (*PGR*, *ESR1*, *OXTR*) (Kotwica *et al.*, 2003; Kobayashi *et al.*, 2016; Kowalik *et al.*, 2016), cell proliferation and adhesion (*MKI67*, *CDH1*) (Ito *et al.*, 2016; Yucer *et al.*, 2021), tight junctions (*OCLN*, *TJP1*) (Celis *et al.*, 2024), oviduct-specific glycoprotein (*OVGP1*) (Bauersachs *et al.*, 2004), and ciliated cell function and differentiation (*LRRC6*, *TUBA1A*, *TUBA1B*, *TUBA1C*, *FOXJ1*) (Kessler *et al.*, 2015; Ito *et al.*, 2016; Lamy *et al.*, 2016; Menjivar *et al.*, 2023b), signifying their aptness to retain a functional *in vivo*-like gene expression signature. The organoids generated in this study and in others (Kessler *et al.*, 2015; Xie *et al.*, 2018; Chang *et al.*, 2020; Ghosh *et al.*, 2020; Lõhmussaar *et al.*, 2020; Yucer *et al.*, 2021; Lawson *et al.*, 2023; Menjivar *et al.*, 2023b; Thompson *et al.*, 2023) contained differentiated epithelial cell types (AC-TUB^+^ and PAX8^+^), along with visible indication of protein markers OVGP1, ZO1, and FOXJ1, highlighting their similarity in both structure and protein expression to intact *in vivo* tissues (Menjivar *et al.*, 2023b). Taken together as a reflection of *in vivo* conditions, this 3D oviductal organoid culture system advances established *in vitro* culture systems by: (i) retention and maintenance of genes governing the function of oviduct epithelium, (ii) facilitating epithelia to regain ciliation, and (iii) maintaining physiological attributes and integrity long term, all of which are not consistently replicated among static 2D systems and are more reflective of *in vivo* conditions.

In cattle, fertilization occurs within the oviduct near the ampulla–isthmus junction, followed by the transient migration of the presumptive zygote toward the uterine lumen as it undergoes its initial cleavage divisions over the next 4 days. These processes are entirely bypassed in current IVF systems, resulting in the loss of maternal communication and the absence of secreted embryotrophic factors around the first major transition occurring following fertilization, a phase marked by dramatic gene expression and epigenetic reprogramming, preceding the MZT/genome activation. Amidst strategies to improve embryo development *in vitro*, efforts to mimic the physiological conditions observed *in vivo* have been more recently enacted through the growing interest in biological information transfer via EV-mediated signaling (Menjivar *et al.*, 2023a). Therefore, we aimed to closely mimic the composition in which embryos would develop naturally *in vivo* from Day 1 to Day 3, through the addition of oEVs from 3D cultured organoids (37°C: N-EVs, 42°C: S-EVs) and OF (Ovi-EVs) *in vitro*. Initially, early insight from the advantageous results of a simplistic IVP co-culture system using oviductal tissue suspensions/conditioned medium (Eyestone and First, 1989), paved way for the large body of literature that would accumulate regarding the unique strategic approach of utilizing oEVs to improve aspects of the *ex vivo* IVP process (Franko and de Almeida Monteiro Melo Ferraz, Marcia, 2024). Aside from those studies focusing on the use of oEVs collected *in vivo*, mechanisms to study cellular communication *ex vivo* rely upon oEVs produced from 2D cell culture models, the current ‘gold standard’. To date, no reported studies have focused on oEVs secreted from niche 3D organoids and their inherent impact on supporting the development of the preimplantation embryo under suboptimal conditions. Similar to previous studies (Stephens *et al.*, 2024), here we visually demonstrate the incorporation of oEVs into the developing embryo, underscoring its receptivity to incorporate and internalize maternal cues. The beneficial effect of oEVs from organoids (N-EVs, S-EVs) and *in vivo* OF (Ovi-EVs) on embryo development was not visible at Day 3, indicated by no difference in the presumptive zygotes’ ability to cleave compared to the non-treated Controls (38.5°C Control and 41°C Control). However, a phenomenon known as the ‘cell block’ or cleavage arrest, observed in cattle (Graf *et al.*, 2014) and human (Braude *et al.*, 1988) embryos, has been reported in association with the MZT occurring at the 8-cell stage. Therefore, these disruptions may not be clearly discernible at Day 3, as this window in time aligns with the determination of the embryos’ cell fate. Moreover, although blastocyst rates do not fully return to baseline levels observed in the Control (38.5°C) following HS exposure, a partial rescuing effect is observed in the treated groups (N-EVs, S-EVs, Ovi-EVs) compared to the 41°C Control, consistent in part with our previous findings (Menjivar *et al.*, 2023a). In addition, developmental plasticity permits the effects of environmental stressors during preimplantation development to subsist altering sex-specific trajectories due to extensive transcriptional dimorphism (Pérez-Cerezales *et al.*, 2018). Taken together, our results may also support such a proposition of transcriptional dimorphism and the extensive receptivity of sex-specific delimitations that perpetually persist from cues (oEVs) the maternal environment contributes during the first week of embryonic development. These findings highlight an inherent future need to understand the EV-mediated cellular and molecular interchange of biological information and its implications in the development of male and female preimplantation embryos preceding the establishment of pregnancy.

Several studies have identified RNA and protein cargo encapsulated within oEVs (Almiñana *et al.*, 2017, 2018), with oEV-associated miRNAs targeting genes enriched in the biological processes that involve embryonic development, embryonic morphology, and implantation. In our co-culture studies with oEVs from organoids (N-EVs, S-EVs) and *in vivo* OF (Ovi-EVs), we observed instigated changes to the pluripotency marker gene expression signatures of individual preimplantation embryos, a preemptive signaling of their undifferentiated state and developmental potential for subsequent lineage specification. Mechanistically, previous studies have linked parallels to their roles as ‘pioneering’ factors binding to silent chromatin as a mechanism facilitating *de novo* gene expression (Soufi *et al.*, 2012), that is in part responsible for mediating the first wave of zygotic genes during the MZT for the establishment of a transient pluripotent state (Lee *et al.*, 2013). In bovine, it is well regarded that the MZT preceding the formation of a blastocyst corresponds to gene expression changes and widespread epigenetic remodeling of DNA methylation, histone modifications, and chromatin structure (Frum and Ralston, 2015). A previous study has shown that the inhibition of embryonic transcription severely impacts H3K27ac remodeling (competitive marks H3K27ac and H3K27me3), leading to the retention of a 4‐cell‐like epigenetic state in 8‐cell embryos (Zhou *et al.*, 2023). Indeed, the co-culture of oEVs from organoids (N-EVs, S-EVs) and *in vivo* OF (Ovi-EVs) revealed increased H3K27ac, indicative of transcriptional activation or chromatin accessibility. Inversely, H3K27me3, which is known to play a major role in the restriction of cellular potency, was reduced in those same blastocysts derived from the co-culture of oEVs, signifying their progressive competency abilities cultured under conditions of stress. In the context of IVF, studies duly suggest that embryos that develop faster, particularly to the blastocyst stage, result in a higher implantation and pregnancy rate compared to slow-developing embryos (Shebl *et al.*, 2021). The dynamic epigenetic reprogramming of H3K27ac and H3K27me3 has been previously reported to be directly connected to the developmental kinetics (Ispada *et al.*, 2021; Ispada and Milazzotto, 2022) and energy metabolism (Ispada *et al.*, 2021; da Fonseca *et al.*, 2023) of bovine embryos, suggesting that in our study, the co-culture of oEVs during IVC may alter the metabolic activity of bovine preimplantation embryos resulting in alterations that accelerate their potential kinetics in establishing a phenotype in line with a competent blastocyst of higher implantation potential. In the same regard, it has been shown previously that embryonic H3K9 acetylation dynamics are stiffly influenced by the pace of the first initial cleavages, where fast-developing embryos are characterized by a progressive decrease in H3K9ac levels from 40 hpi to the blastocyst stage (Ispada *et al.*, 2021). Furthermore, these data suggest that the internalization of oEVs poses an eminent system of biological information transfer capable of impacting the regulation of post-translation histone modifications amid the early developing embryo.

Our current understanding of the impact of deficient genetic information transfer between maternal tissues and the developing preimplantation embryo *in vitro* is limited. The discovery of EVs and their involvement in intercellular communication mediated by cargo molecules such as the functional repertoire of EV-associated miRNA profiles that has propagated a whole new level of complexity in understanding such a molecular dialogue. Recent evidence has shown that EV-miRNA cargo derived from 3D systems is more similar to *in vivo* signatures than 2D cell monolayers (Thippabhotla *et al.*, 2019), a gap that has not been attempted for comparing oEVs (3D, 2D, and *in vivo*). An integrated meta-analysis comparing oEV-associated miRNAs from our previously published 3D study (Menjivar *et al.*, 2023b), 2D (Reshi *et al.*, 2023; Hamdi *et al.*, 2024), and *in vivo* OF (Almiñana *et al.*, 2018; Hamdi *et al.*, 2021; Mazzarella *et al.*, 2021; Leal *et al.*, 2022), enabled the identification and interpretation of environmental influences reflected in distinct miRNA fingerprints. A unique signature of miRNAs (bta-miR-122, bta-miR-192, bta-miR-148a, bta-miR-30d, and bta-miR-10b) was found to be mutually expressed in oEVs from organoids (N-EVs, S-EVs) and *in vivo* OF (Ovi-EVs), potentially in part responsible in the functional regulation of essential genes governing preimplantation embryonic development. Notably, the overwhelming significance and regulatory role of oEV-encapsulated bta-miR-148a is supported by its validated *DNMT1* target (Wang *et al.*, 2017). In the same study, the overexpression of bta-miR-148a in SCNT embryos showed significant increases in blastocyst formation, total cell numbers, altered H3K9ac levels, and enhanced pluripotency markers *OCT4* and *NANOG*, all of which support the results found in this co-culture study with oEVs. Additionally, studies involving exosome-associated hsa-miR-30d and its acting role as a transcriptomic modifier have been shown in relation to its internalization amid the TE of mouse embryos, augmenting the expression of genes regulating cellular adhesion (*ITGB3*, *ITGA7*, and *CDH5*), resulting in a notable increase in embryo adhesion (Vilella *et al.*, 2015). The alignment of the validated regulatory effects of such maternally shuttled oEV-miRNAs suggests that maternal-embryonic communication within the oviduct is in part essential to an embryo’s decision in cell fate and its ability in establishing competency prior to implantation amidst a receptive endometrium. These results suggest that the developmental plasticity of oviductal epithelium cultured as 3D organoids in part favors an EV-miRNA crossover with that found *in vivo*, and that bta-miR-122, bta-miR-192, bta-miR-148a, bta-miR-30d, and bta-miR-10b underscore independent candidates that may labor synergistically as potential key mediators during EV-mediated signaling and communication of maternal tissues and the developing embryo.

Our study does incur possible limitations to consider upon interpretation that largely stem from the orientation of polarized cells in organoids and the use of basolateral (N-EVs and S-EVs) versus apical secretions (Ovi-EVs), which would occur *in vivo*. Composition differences do in fact persist between intra/extra-organoid fluid, likely containing in part some apical versus basolateral secreted oEVs (Simintiras *et al.*, 2021). However, studies focused exclusively on understanding how polarized cells communicate through EVs in a more physiologically relevant manner are extensively understudied. We recognize that polarity-reversed organoids (apical-out) may be a furthered attempt to better model the subsequent aforementioned goals of the embryo–oviduct dialog. Whether true cargo-specific differences in EVs released exclusively from apical surfaces in the context of this work incur inherent molecular and functional differences in developing embryos needs further exploration.

Taken together, our findings not only deepen our understanding of the EV-mediated signaling between the oviduct and the preimplantation embryo but also highlight the potential utilization of oviductal organoids as a non-invasive model system for elucidating the molecular dialogue during preimplantation embryonic development *in vivo* and under various pathological conditions. Future studies will aim to dissect the differences among oEVs secreted from hormonally-stimulated oviductal organoids and those derived from polarity-reversed organoids, and to further expand upon approaches involving a more directed co-culture of oviductal organoids with developing preimplantation embryos, an approach partially explored using an oviduct epithelial spheroid model (Pranomphon *et al.*, 2024a,b). Ultimately, our methodology, supported by functionally validated studies, identifies oEVs derived from 3D organoids as a promising novel EV-based treatment to enhance embryonic development and quality in animal *in vitro* ART systems with the potential translational relevance to human IVF clinics.

## Supplementary Material

hoaf076_Supplementary_Data

## Data Availability

Reasonable requests for further information and resources should be directed to, and will be fulfilled by, the corresponding author, Dawit Tesfaye (dawit.tesfaye@colostate.edu). Data underlying this article analyzes existing, publicly available data accessible in the Gene Expression Omnibus (GEO): GSE221895 and GSE110443, as well as the Sequence Read Archive (SRA): PRJNA928588. Additional information regarding the studies indicated in Table 1 were requested and provided by the respective lead contacts of the studies. Additional information required to reanalyze the data reported in this paper is available from the corresponding author upon request.
