# Supplementary figures and images for: Organoids simulating the bovine oviduct mediate the embryo–maternal interface via extracellular vesicle-transmitted signaling
Source: Hum Reprod Open. 2025 Dec 5;2026(1):hoaf076. doi: 10.1093/hropen/hoaf076 (PMC12774516; doi:10.1093/hropen/hoaf076)

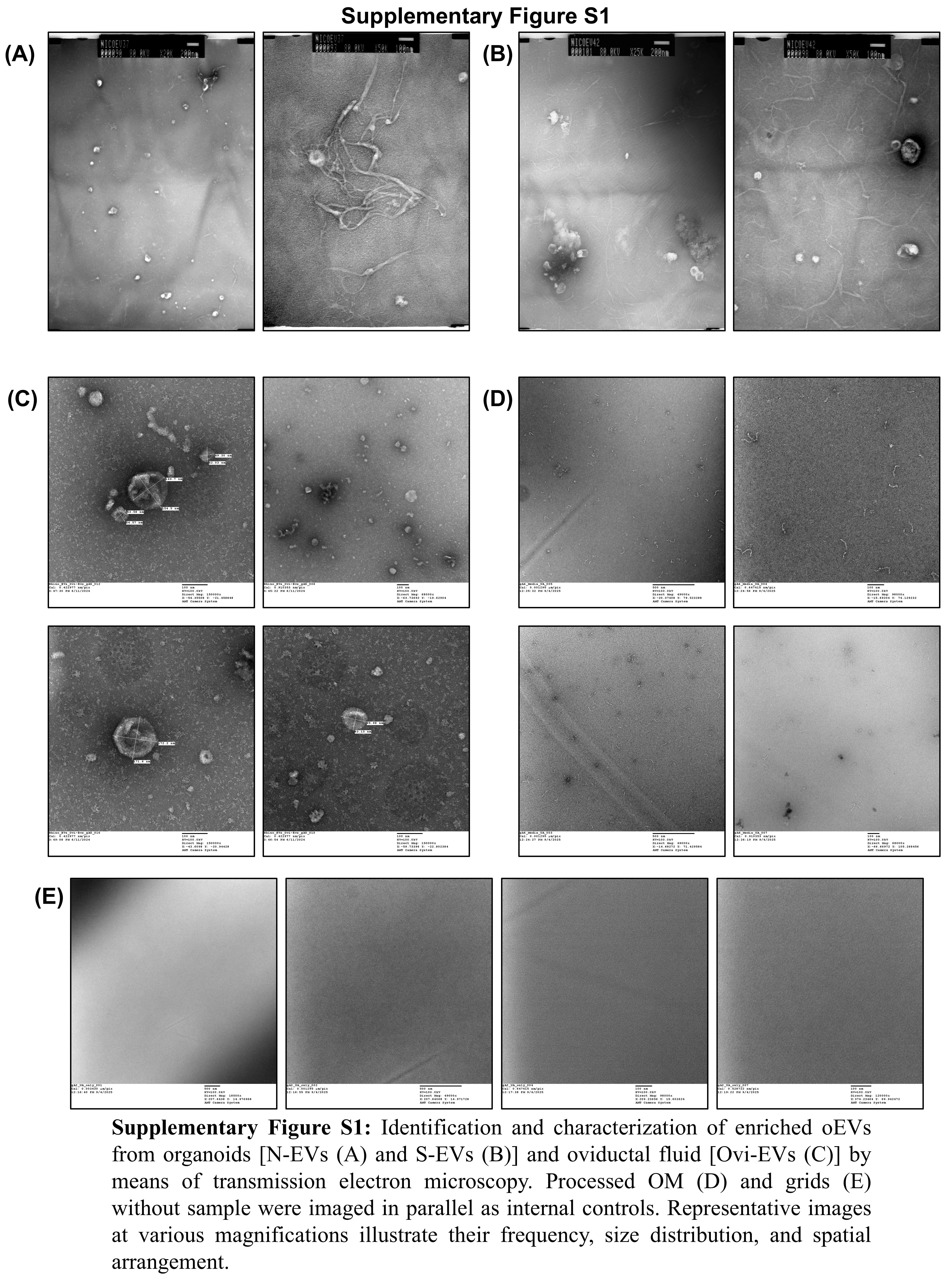

Supplement: hoaf076_Supplementary_Data [file hoaf076_supplementary_data.zip › Supplementary Figure S1.tif]

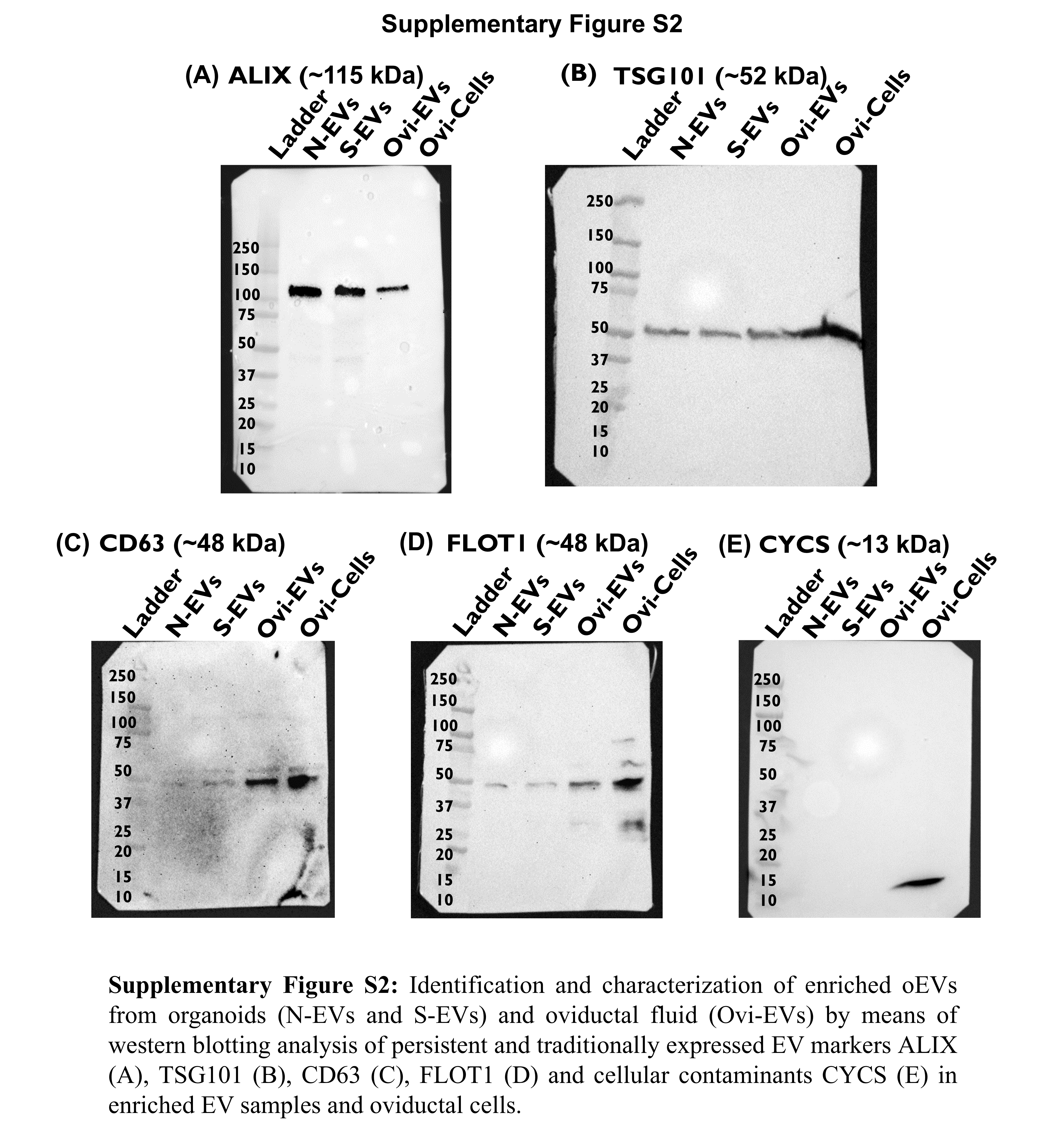

Supplement: hoaf076_Supplementary_Data [file hoaf076_supplementary_data.zip › Supplementary Figure S2.tif]

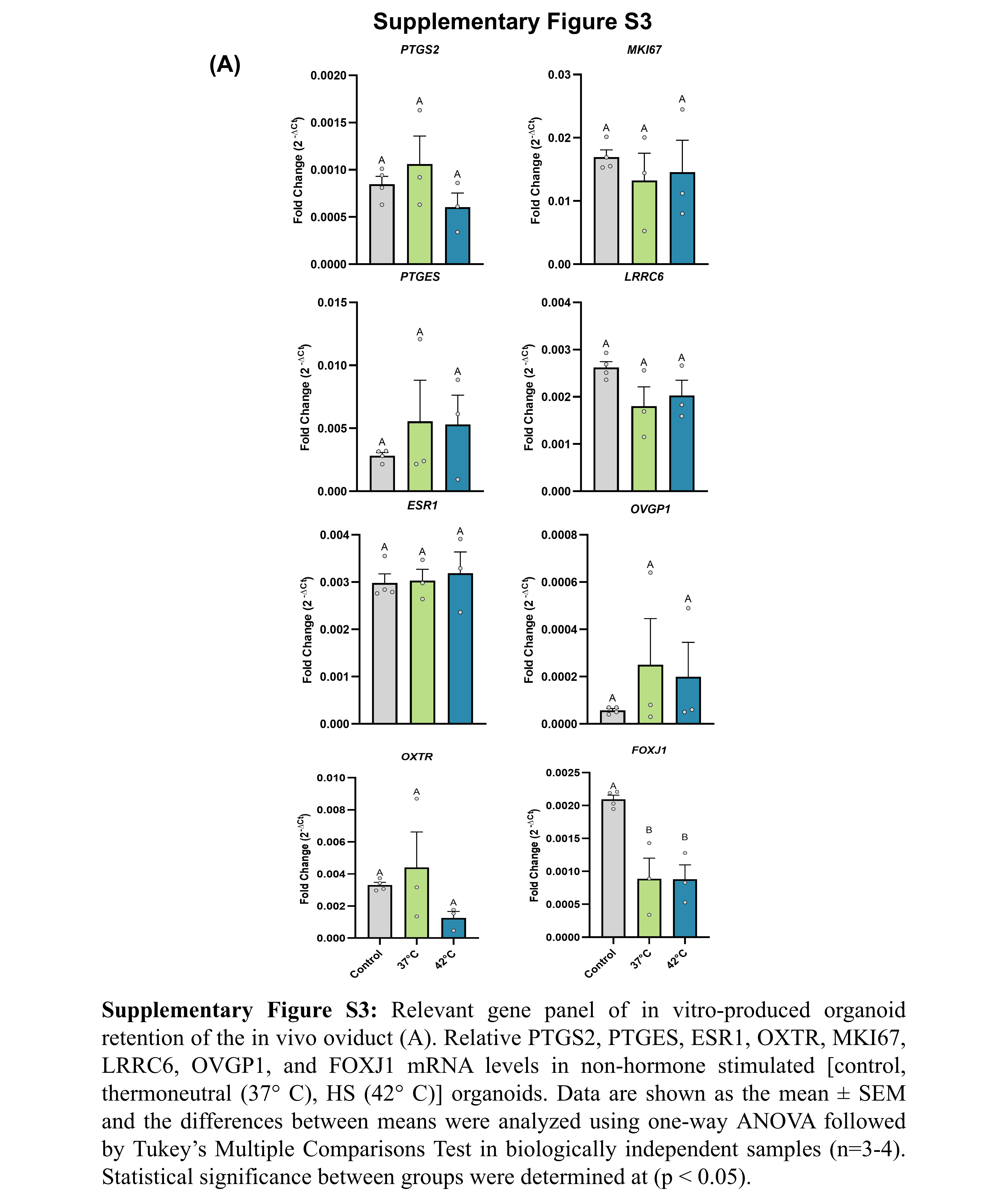

Supplement: hoaf076_Supplementary_Data [file hoaf076_supplementary_data.zip › Supplementary Figure S3.tif]
